# Extensive editing of cellular and viral double-stranded RNA structures accounts for innate immunity suppression and the proviral activity of ADAR1^p150^

**DOI:** 10.1371/journal.pbio.2006577

**Published:** 2018-11-29

**Authors:** Christian K. Pfaller, Ryan C. Donohue, Stepan Nersisyan, Leonid Brodsky, Roberto Cattaneo

**Affiliations:** 1 Department of Molecular Medicine, Mayo Clinic, Rochester, Minnesota, United States of America; 2 Mayo Clinic Graduate School of Biomedical Sciences, Rochester, Minnesota, United States of America; 3 Tauber Bioinformatics Research Center, University of Haifa, Haifa, Israel; 4 Lomonosov Moscow State University, Moscow, Russia; New York University, United States of America

## Abstract

The interferon (IFN)-mediated innate immune response is the first line of defense against viruses. However, an IFN-stimulated gene, the adenosine deaminase acting on RNA 1 (ADAR1), favors the replication of several viruses. ADAR1 binds double-stranded RNA and converts adenosine to inosine by deamination. This form of editing makes duplex RNA unstable, thereby preventing IFN induction. To better understand how ADAR1 works at the cellular level, we generated cell lines that express exclusively either the IFN-inducible, cytoplasmic isoform ADAR1^p150^, the constitutively expressed nuclear isoform ADAR1^p110^, or no isoform. By comparing the transcriptome of these cell lines, we identified more than 150 polymerase II transcripts that are extensively edited, and we attributed most editing events to ADAR1^p150^. Editing is focused on inverted transposable elements, located mainly within introns and untranslated regions, and predicted to form duplex RNA structures. Editing of these elements occurs also in primary human samples, and there is evidence for cross-species evolutionary conservation of editing patterns in primates and, to a lesser extent, in rodents. Whereas ADAR1^p150^ rarely edits tightly encapsidated standard measles virus (MeV) genomes, it efficiently edits genomes with inverted repeats accidentally generated by a mutant MeV. We also show that immune activation occurs in fully ADAR1-deficient (ADAR1^KO^) cells, restricting virus growth, and that complementation of these cells with ADAR1^p150^ rescues virus growth and suppresses innate immunity activation. Finally, by knocking out either protein kinase R (PKR) or mitochondrial antiviral signaling protein (MAVS)—another protein controlling the response to duplex RNA—in ADAR1^KO^ cells, we show that PKR activation elicits a stronger antiviral response. Thus, ADAR1 prevents innate immunity activation by cellular transcripts that include extensive duplex RNA structures. The trade-off is that viruses take advantage of ADAR1 to elude innate immunity control.

## Author summary

The innate immune response is a double-edged sword. It must protect the host from pathogens while avoiding accidental recognition of “self” molecular patterns, which can lead to autoimmune reactions. Double-stranded RNA is among the most potent inducers of cellular stress and interferon responses. We characterize here a mechanism that prevents autoimmune activation and show that an RNA virus, measles virus, can exploit it to elude innate immune responses. This mechanism relies on the enzyme adenosine deaminase acting on RNA 1 (ADAR1), which converts adenosine residues within duplex RNA structures to inosine. We identify duplex RNA structures in the 3′ untranslated regions of over 150 cellular transcripts and show that they are heavily edited in ADAR1-expressing cells. We detect the same type of editing in duplex RNA–forming defective genomes accidentally generated by measles virus. Loss of RNA editing causes strong innate immune responses and is detrimental to viral replication. Thus, by keeping the amount of duplex RNA in cells below an immune activation threshold, ADAR1 prevents autoimmunity while also favoring pathogens.

## Introduction

The innate immune response is the first line of defense against viruses [1]. This response, which must tolerate self, is based on the concerted action of interferon (IFN)-stimulated gene (ISG) products. Yet one of these, the adenosine deaminase acting on RNA 1 (ADAR1), has a key role in suppressing IFN signaling [2]. Here, we seek to characterize how ADAR1 functions. ADARs convert adenosine residues (C6 position) to inosine in double-stranded RNA (dsRNA), a process known as A-to-I editing [3,4]. There are three mammalian *ADAR* genes, but only ADAR1 and ADAR2 proteins edit RNA in vitro [5]. ADAR2 modifies the coding capacity of specific transcripts and the biological function of the corresponding proteins [5]. ADAR1 editing is less targeted and very extensive in many tissues, as revealed by next-generation sequencing [6,7]. However, the significance of this massive editing is still largely unexplored [8].

Mammalian ADAR1 is expressed in two isoforms: constitutive ADAR1^p110^ and IFN-inducible ADAR1^p150^ [9]. Both enzymes consist of a carboxyl-terminal deaminase domain, three consecutive dsRNA binding motifs (RNA-binding motifs I–III [RBM_I–III_]), and an amino-terminal Z-DNA binding domain (Zβ). ADAR1^p150^ includes an additional amino-terminal Z-DNA binding domain (Zα) [10]. Whereas ADAR1^p110^ is predominantly located in nuclei, ADAR1^p150^ exhibits nucleocytoplasmic distribution through a nuclear export signal in the Zα domain [11,12].

ADAR1 has a key role in suppressing IFN responses [2]. Knock-out of the *Adar* locus is embryonically lethal in mice [13] but can be rescued by the additional disruption of three genes controlling the innate immune response to dsRNA: melanoma differentiation–associated gene 5 (MDA-5) [14,15], mitochondrial antiviral signaling protein (MAVS, also known as mitochondrial IFN-beta promoter stimulator-1 [IPS-1], virus-induced signaling adapter [VISA], CARD adapter inducing interferon-beta [CARDIF]) [16], or the 2′-5′-oligoadenylate-synthetase (2′-5′-OAS)-dependent RNase L [17]. MAVS is a mitochondria-associated adapter required for IFN induction by retinoic acid–inducible gene I (RIG-I)-like receptors (RLRs) RIG-I and MDA-5 [18]. Moreover, human *ADAR1* mutations are associated with autoimmune diseases like Aicardi-Goutières Syndrome (AGS type 6 [AGS6]) [19] and dyschromatosis symmetrica hereditaria (DSH1) [20]. Altogether, these observations suggest that ADAR1 and these antiviral response genes have opposite effects on the control of innate immune responses to endogenous duplex RNA.

Indeed, ADAR1 can be proviral: it favors the replication of positive-strand RNA viruses such as yellow fever virus, Venezuelan equine encephalitis virus, and Chikungunya virus [21] and of negative-strand RNA viruses including measles virus (MeV) [22,23]. On the other hand, ADAR1 can damage viral RNA genomes by introducing large clusters of mutations, read as A-to-G (A>G) or U-to-C (U>C), depending on the strand edited [24,25].

We have recently shown that extensive ADAR1 editing occurs in defective interfering (DI) MeV RNAs generated during replication of a mutant MeV unable to express C protein (MeV-C^KO^) [22,26]. These DI RNAs can form panhandle duplex RNA structures if not properly encapsidated. Since C protein controls viral polymerase fidelity, MeV-C^KO^ generates elevated levels of DI RNA and induces strong innate immune responses [22,26–29]. These responses involve activation of protein kinase R (PKR), which leads to translational arrest [28] and formation of stress granules [22,30].

ADAR1, on the other hand, interferes with the immune activation by viruses [23,30,31]. Here, we take advantage of two recombinant MeVs, C^KO^(GFP) and its isogenic parental virus vac2(GFP), and of a newly generated set of HeLa cells expressing different ADAR1 isoforms to characterize the endogenous and viral duplex RNA that activate innate immunity.

## Results

### Innate immunity is activated in ADAR1-defective cells

To better characterize the mechanisms of action of the two isoforms, we targeted exon 2 of the *ADAR* locus (S1A Fig) and generated selectively ADAR1^p150^-deficient (p150^KO^) and fully ADAR1-deficient (ADAR1^KO^) HeLa cells. Multiple independent clones were recovered for each cell line and analyzed by western blot (S1B Fig). Genetic alterations causing the knock-out were deduced from RNA sequencing (RNAseq) data for 2 clones (p150^KO^-B13 and ADAR1^KO^-E7) (S1C Fig). In addition, we complemented ADAR1^KO^ cells with lentiviral vectors expressing wild-type ADAR1^p150^ (p150wt_LV_) or catalytically inactive ADAR1^p150^ (p150mut_LV_) [30,32]. We confirmed that both proteins had the expected cytoplasmic localization (S1D Fig). We also verified that knock-out of ADAR1 had no effect on the expression of ADAR2, which was predominantly found in nuclear extracts as expected (S1E Fig).

To assess whether ADAR1 deficiency affects cell viability or division rate, we performed a time course experiment comparing parental HeLa cells with p150^KO^ (B13) and ADAR1^KO^ (E7) cells (S2 Fig). For this, cells were stained with CellTrace Violet, and, at 1-d intervals, levels of live, apoptotic, and dead cells were determined (S2A Fig). ADAR1^KO^ cells showed slightly increased numbers of apoptotic and dead cells as compared to HeLa and p150^KO^ cells (S2B Fig and S1 Data). The division rate of each cell population was determined by loss of CellTrace Violet fluorescence (S2C Fig), using the signal of HeLa cells to determine gates for each cell division (S2C Fig, dashed lines). p150^KO^ and ADAR1^KO^ cells showed no difference to HeLa cells at 0 and 24 h but had more cells with lagging division rates at later time points, which was most pronounced in apoptotic and dead cell populations. ADAR1^KO^ cells and, to a lesser extent, p150^KO^ cells had higher fractions of cells in lower division rates as compared to HeLa cells (S2D Fig). From this data, we deduced the time required for 50% of the cell population to divide (division time 50 [DT_50_]) (S2C Fig, dotted black lines) and calculated the average division rate, which was between 24 and 26 h for each cell line (S2D Fig). Thus, p150^KO^ and ADAR1^KO^ cells have similar division rates as parental HeLa cells and similar or slightly reduced viability.

**Fig 1:**
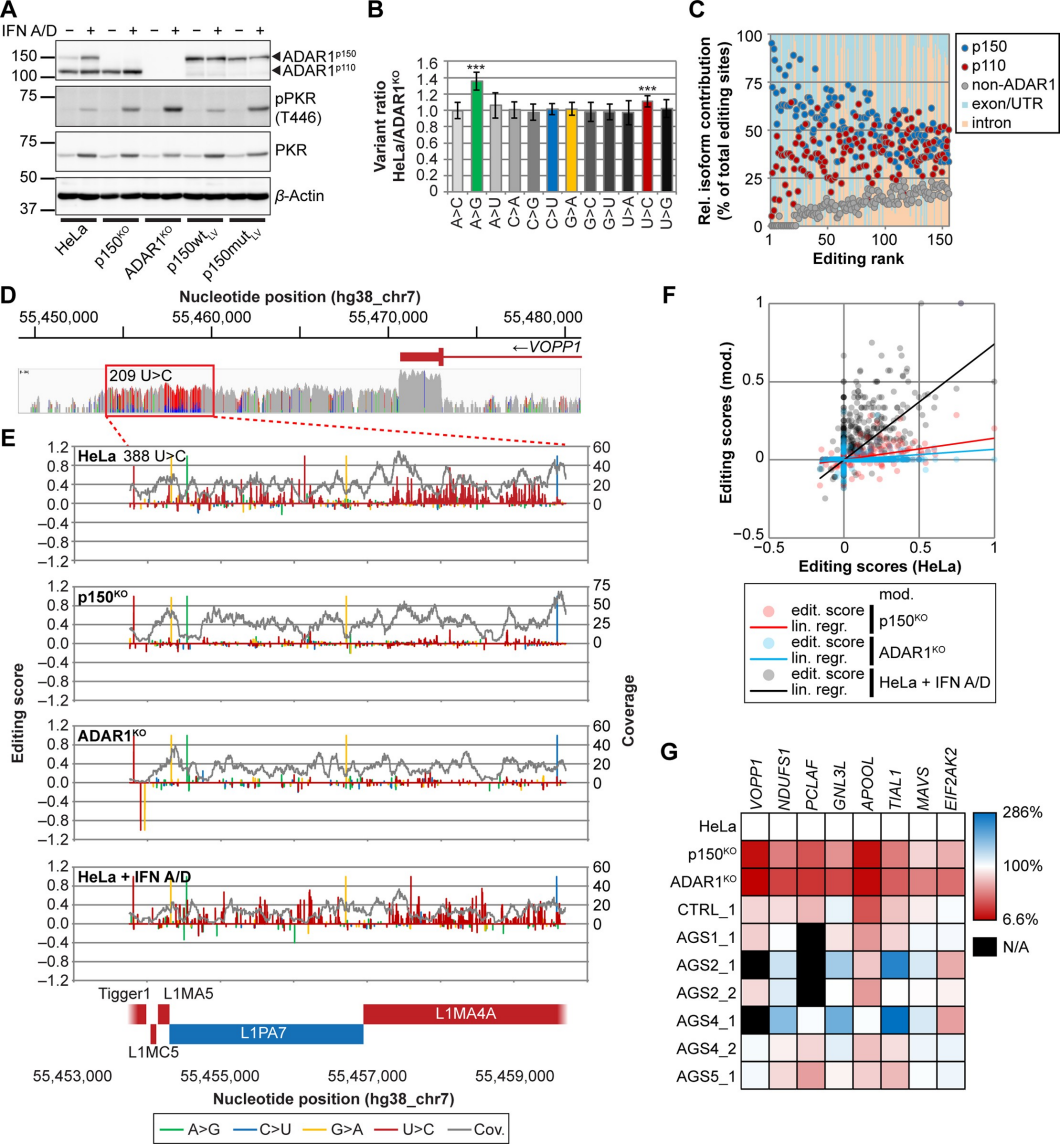
ADAR1 edits cellular transcripts forming duplex RNA structures. (A) Western blot analysis of untreated and IFN A/D–treated ADAR1-modified cells. Cells were treated with 1,000 U/ml IFN A/D for 24 h. (B) Ratios of absolute numbers of identified variants in HeLa and ADAR1^KO^ cells. A>G and U>C variants show higher enrichment in HeLa cells. Average and standard deviation of numbers of variants are indicated. *P* values of each variant were determined by one-sample, two-tailed Student’s *t* test against an expected value of 1 (***, *P* ≤ 0.0001). Underlying values can be found in S1 Data. (C) Relative contribution of ADAR1 isoforms (p150 and p110) to editing in the top 156 genes and correlation with editing in exons/UTRs or introns. Values are calculated based on the number of editing sites detected by GIREMI in HeLa, p150^KO^, and ADAR1^KO^ cells. Non-ADAR1, percentage of editing sites remaining in ADAR1^KO^ cells. (D) Coverage plot of the 3′ UTR region of *VOPP1*. The region in the red box shows strong enrichment of U>C transitions. Nucleotide positions with variant frequencies ≥10% are color-coded: A, green; C, blue; G, orange; U, red. (E) Editing score analysis of the boxed region of (D) in HeLa, p150^KO^, ADAR1^KO^, and HeLa cells treated with 1,000 U/ml IFN A/D (top to bottom). Gray line indicates total coverage (“Cov.”) in the region. Repetitive sequences are indicated at the bottom: positive sense in blue and negative sense in red. (F) Correlation of editing scores of the *VOPP1*-region in HeLa + IFN A/D against untreated HeLa cells (gray dots, black line), HeLa versus p150^KO^ (red dots and line), and HeLa versus ADAR1^KO^ (blue dots and line). (G) Relative editing rates in cell lines and primary RNAseq data. Editing rates are normalized to coverage and length of the analyzed regions. Editing rate of each gene in HeLa cells is set to 100%. ADAR1, adenosine deaminase acting on RNA 1; ADAR1^KO^, fully ADAR1-deficient; GIREMI, Genome-independent Identification of RNA Editing by Mutual Information; IFN A/D, recombinant type-I interferon-alpha; lin. regr., linear regression; N/A, no RNAseq data available because of low or no coverage; p150^KO^, selectively ADAR1^p150^-deficient; p150mut_LV_, catalytically inactive ADAR1^p150^; p150wt_LV_, wild-type ADAR1^p150^; PKR, protein kinase R; pPKR, phospho-PKR; RNAseq, RNA sequencing; UTR, untranslated region; *VOPP1*, *vesicular*, *overexpressed in cancer*, *prosurvival protein 1*.

We also asked whether innate immunity is activated in ADAR1-deficient cells. Indeed, treatment with recombinant type-I IFN-alpha (IFN A/D) resulted in stronger PKR activation in p150^KO^ and ADAR1^KO^ cells compared to parental HeLa or p150wt_LV_ cells (Fig 1A). Since PKR is activated upon dsRNA binding, we think that endogenous transcripts forming dsRNA structures cause this activation. Catalytically inactive ADAR1^p150^ did not fully suppress PKR activation (Fig 1A). Thus, ADAR1^p150^ but not ADAR1^p110^ can interfere with recognition of endogenous dsRNA by PKR.

### Differential editing of nuclear and cytoplasmic RNA by the two ADAR1 isoforms

To gain insights on the cellular transcripts that may activate PKR if left unedited by ADAR1, we used deep sequencing to characterize the total transcriptomes of HeLa, p150^KO^, and ADAR1^KO^ cells. To identify potential editing sites, we adopted the Genome-independent Identification of RNA Editing by Mutual Information (GIREMI) method (S3A Fig) [33]. As expected, we detected reduced frequencies of A>G and U>C transitions in ADAR1^KO^ cells (26,334 A>G sites) compared to HeLa cells (35,403 A>G sites), whereas the ratios of C>U and G>A transitions and of all transversions were unchanged (S3B Fig and S1 Data). Symmetrically, A>G and U>C transitions were increased about 1.4- and 1.15-fold, respectively, in HeLa cells compared to ADAR1^KO^ cells (Fig 1B, S3C Fig, and S1 Data). This is consistent with the enzymatic activity of ADAR1, which results in A>G and U>C transitions, depending on the strand analyzed. More than half of editing sites were located in intronic sequences, whereas exons and untranslated regions (UTRs) accounted for about 25% of A>G events (S3D Fig and S1 Data). This ratio remained unchanged in p150^KO^ and ADAR1^KO^ cells despite the overall reduction of the number of A>G events in ADAR1^KO^ cells, reflecting a high fraction of A>G events detected by GIREMI in the “junk DNA” genome fragments.

Since other studies indicate that ADAR1 editing preferentially occurs in Alu elements [6,34,35], we validated our approach by testing this correlation. We found that about 25% of all A>G transitions in HeLa cells are associated with retrotransposable elements. In particular, Alu elements formed the largest fraction of edited elements (over 75%), followed by long interspersed nuclear element (LINE) L1 elements and 7SL RNA (S3E Fig and S1 Data), which is consistent with previous analyses [6,34,35].

We then asked how the sites identified by GIREMI may get edited by ADAR1 within individual transcripts. We identified the most-edited transcripts based on four inclusion criteria (see Methods section) (S1 Table). In HeLa cells, within the top 156 transcripts, half of the editing sites were in introns, and the others in were in exons and UTRs (S4A Fig, left column and S1 Data). With loss of the ADAR1^p150^ isoform (p150^KO^), remaining editing was more prevalent in introns (S4A Fig, middle column and S1 Data). This is consistent with intron editing by nuclear ADAR1^p110^, whereas ADAR1^150^ editing occurs mainly in exons and UTRs. On the other hand, there was no preferential editing of specific transposable elements by either ADAR1 isoform (S4B Fig and S1 Data).

We also noted that the highest-ranking genes in our data set were predominantly edited by ADAR1^p150^ (Fig 1C, blue dots), and editing mostly occurred in exons/UTRs (Fig 1C, light blue shading). In contrast, lower-ranking genes were equally targeted by both ADAR1 isoforms (Fig 1C, blue and red dots), and editing occurred at higher frequencies in intronic regions (Fig 1C, orange shading). These data are consistent with ADAR1^p150^ being mainly responsible for editing of cytoplasmic transcripts.

### ADAR1 editing patterns of HeLa cells and primary tissues are consistent

We then refined analyses of editing within individual transcripts by constructing coverage plots and comparing their ADAR1 editing levels in HeLa, p150^KO^, and ADAR1 ^KO^ cell lines. Fig 1D shows coverage plots of the 3′ UTR of the *VOPP1* (*vesicular*, *overexpressed in cancer*, *prosurvival protein 1*) transcript, which had the highest A>G transition differential. Whereas GIREMI detected 58 editing sites in a 6-kb region, the coverage plot was more sensitive, detecting 209 U>C transitions with >10% conversion rate (Fig 1D, red box). In a further analytical refinement, we developed a method to compensate for sequencing mistakes, which returned a positive editing score for 388 sites in this region (Fig 1E, top diagram). The edited region overlaps with two inverted LINE elements (Fig 1E, bottom). These inverted elements are predicted to form a nearly 3-kbp duplex secondary structure (not shown). Editing score analyses of p150^KO^ and ADAR1^KO^ cells detected only background levels of U>C transitions (Fig 1E, second and third panel, and Fig 1F, red and blue dots and lines). In addition, editing scores were unaltered upon IFN A/D treatment of HeLa cells (Fig 1E, fourth panel, and Fig 1F, gray dots and black line).

To address the biological relevance of our HeLa cell–based observations, we repeated the GIREMI analyses with RNAseq data sets from human donors. Since HeLa cells are derived from cervical carcinoma [36], we repeated GIREMI analyses with data sets derived from primary cervical stromal cells (CSCs; Gene Expression Omnibus [GEO]: GSE99392) [37] (S1 Table and S4C Fig). The total number of identified editing sites was generally lower in the primary data sets (S4C Fig and S1 Table). However, the list of edited transcripts derived from primary data sets largely overlapped with the list derived from HeLa cells, and the affected regions were identical (S1 Table). Next, we analyzed primary human fibroblast RNAseq data of a healthy individual (CTRL_1) and several patients with ADAR1-sufficient AGS (GEO: GSE57353) [38]. Similarly as with the other data sets, GIREMI identified editing in the same transcripts as in HeLa cells (S1 Table and S4C Fig), indicating that ADAR1 editing is similar in different cell types. In more detail, the characteristic HeLa cell ADAR1 editing pattern of the *VOPP1* transcript was maintained in all five primary human samples with adequate sequence coverage (S5A–S5E Fig), and the same applied to editing scores (S5F–S5J Fig). Editing patterns from individuals were similar but not identical: more than 100 editing sites defined in HeLa cells were found in all five human samples, whereas more than 200 were found in 1–4 samples (S5K and S5L Fig and S1 Data).

We selected 8 transcripts from top, center, and bottom regions of S1 Table and compared editing frequencies in HeLa cells with those of primary human data sets (Fig 1G). These analyses indicate that, although differences between individuals exist, editing frequencies for these transcripts remain similar in the human population. Altogether, ADAR1 editing in HeLa cells generally compares well with editing in primary human samples, but individual editing patterns can differ considerably.

### ADAR1 edits short interspersed nuclear elements (SINEs) in primates and rodents

**Fig 2:**
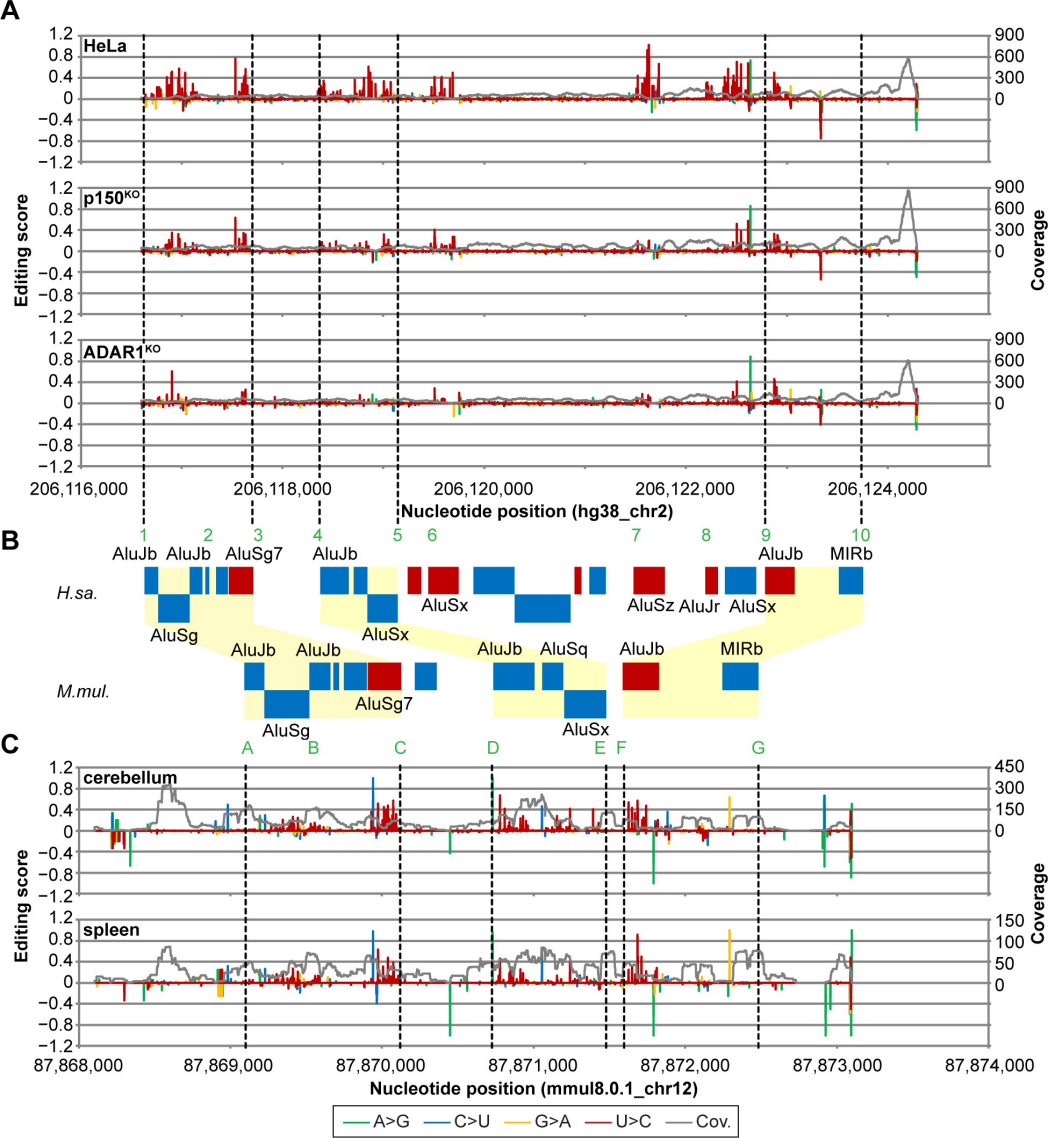
ADAR1-editing patterns are conserved in different tissues of primates. (A) Editing scores in the 3′ UTR region of *NDUFS1* (antisense gene) in HeLa, p150^KO^, and ADAR1^KO^ cells (from top to bottom). (B) Organization of transposable elements in the 3′ UTR of *NDUFS1*. Positive-sense elements are shown in blue, negative-sense elements in red. Top half shows the human gene (*H*.*sa*.), bottom half the macaque (*M*.*mul*.) gene. Yellow highlighted regions are conserved across species. Green numbers and letters refer to approximate positions in secondary structures in S6A Fig and S6B Fig. (C) Editing scores in the 3′ UTR region of macaque *NDUFS1* derived from RNAseq data of cerebellum and spleen [39]. ADAR1, adenosine deaminase acting on RNA 1; ADAR1^KO^, fully ADAR1-deficient; *H*.*sa*., *Homo sapiens*; *M*.*mul*., *M*. *mulatta*; *NDUFS1*, *NADH*:*ubiquinone oxidoreductase core subunit S1*; p150^KO^, selectively ADAR1^p150^-deficient; RNAseq, RNA sequencing; UTR, untranslated region.

Having identified inverted repetitive elements as the primary target within ADAR1-edited transcripts, we asked whether homologous transcripts of different species include repetitive elements potentially forming duplex RNA structures. Towards this, we analyzed data sets from rhesus macaques (*Macaca mulatta*) [39] and mice (*Mus musculus*) [14]. Fig 2A illustrates one example of evolutionarily conserved ADAR1 editing. The 3′ UTR of the human *NADH*:*ubiquinone oxidoreductase core subunit S1* (*NDUFS1*) transcript includes 20 transposable elements in a complex arrangement (Fig 2B, top half). Eleven of these elements are extensively edited by ADAR1, as indicated by the local concentration of many high editing score positions (Fig 2A). All these high-editing regions are predicted to form duplex structures (S6A Fig).

In the corresponding macaque transcript, the repetitive elements arrangement is simpler, but three groups of Alu repeats are conserved (Fig 2B, bottom half). Editing in these elements is conserved between humans (Fig 2A) and macaques (Fig 2C). The additionally inserted Alu elements in the human transcript change the Alu–Alu duplex formation (S6A Fig) as compared to the macaque transcript (S6B Fig), which impacts the specific ADAR1 editing frequencies in each element. Editing is conserved across monkey tissues, including cerebellum, spleen (Fig 2C), heart, kidney, and lung (S6C Fig, top to bottom). Other transcripts, including *APOOL*, *GNL3L*, *TIAL1*, and *EXOSC2*, were similarly edited in human and macaque samples.

Alu elements are very abundant in humans and other primates [35,40] but not in rodents [41]. Nevertheless, we asked whether ADAR1 editing is conserved across orders. Comparison of the 156 ADAR1-edited human transcripts with 129 ADAR1-edited murine transcripts [14] identified 7 homologous genes: *GNL3L*, *XPNPEP3*, *MAD2L1*, *BRI3BP*, *MALT1*, *DFFA*, and *RBBP4*. Editing in murine transcripts occurs in Alu-lineage repetitive elements B1, B3, and B4 [41]. For example, ADAR1 edits the human *BRI3BP* transcript in two inverted Alu elements (S7A Fig) predicted to form duplex RNA (S7B Fig). Analogously, ADAR1 edits the murine *Bri3bp* transcript within B1 elements (S7C Fig) forming duplex RNA (S7D Fig). Thus, ADAR1 editing of certain SINEs is conserved between rodents and primates, and higher ADAR1 editing prevalence in humans correlates with selective amplification of Alu elements.

### Almost 1% of cellular transcripts are extensively ADAR1 edited

**Fig 3:**
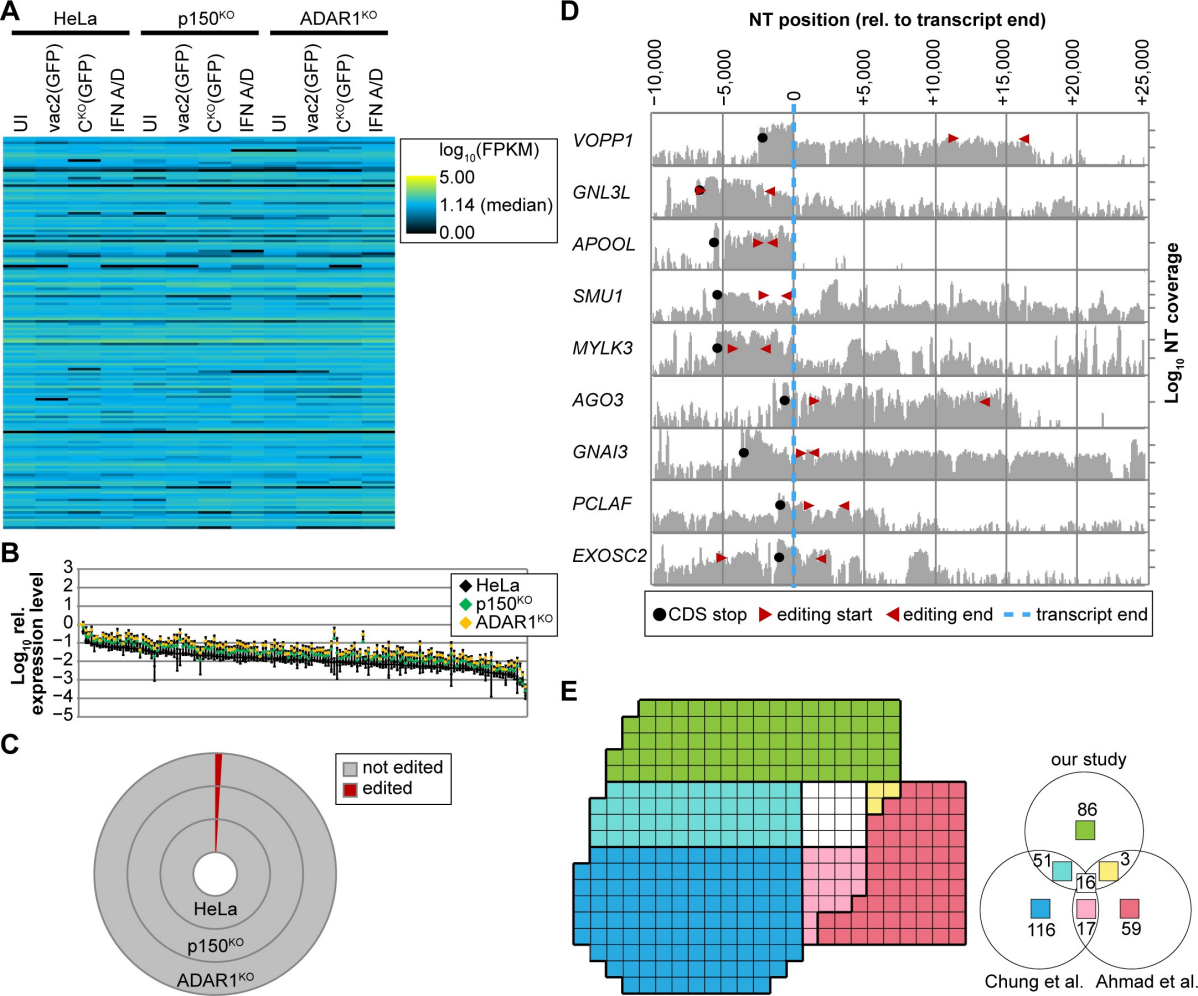
ADAR1 editing in cellular transcripts sets an immune-activation threshold for dsRNA. (A) Quantification of ADAR1-edited transcripts (order as in S1 Table) in HeLa, p150^KO^, and ADAR1^KO^ cells. Four conditions were analyzed for each cell type: UI, infected with MeV-vac2(GFP), infected with MeV-C^KO^(GFP) (both at MOI = 3 for 24 h), or treated with IFN A/D (1,000 U/ml for 24 h). Heatmap shows log_10_ FPKM values from RNAseq analysis. (B) Normalized expression levels of ADAR1-edited transcripts relative to GAPDH levels. Median levels for the four conditions described in (A) are shown. Error bars indicate 95% confidence interval. (C) Proportion of ADAR1-edited (red) and nonedited (gray) in HeLa, p150^KO^, and ADAR1^KO^ cells. (D) Coverage plots and location of ADAR1 editing relative to annotated transcript 3′ end (blue dashed line). Editing occurs between red triangles. Black dot indicates CDS stop codon. (E) Comparison of ADAR1-edited transcripts identified by us (green, cyan, yellow, white), Chung and colleagues [34] (blue, cyan, magenta, white), and Ahmad and colleagues [42] (red, magenta, yellow, white). Each transcript is represented by a single tile. The total numbers in each group (unique, shared by two or three independent studies) are indicated in the legend to the lower right. ADAR1, adenosine deaminase acting on RNA 1; ADAR1^KO^, fully ADAR1-deficient; CDS, coding sequence; dsRNA, double-stranded RNA; FPKM, fragments per kilobase of transcript per million mapped reads; GAPDH, glyceraldehyde 3-phosphate dehydrogenase; IFN A/D, recombinant type-I interferon-alpha; MOI, multiplicity of infection; NT, nucleotide; p150^KO^, selectively ADAR1^p150^-deficient; RNAseq, RNA sequencing; UI, uninfected.

Having identified more than 150 ADAR1-edited transcripts, we sought to estimate their expression levels and thus the amount of dsRNA present in a cell. For this, we relied on RNAseq-based transcript quantification in HeLa, p150^KO^, and ADAR1^KO^ cells, using four conditions (uninfected, vac2[GFP]-infected, C^KO^[GFP]-infected, and IFN A/D-treated) for each cell line (Fig 3A and S8A Fig).

We determined values for the fragments per kilobase of transcript per million mapped reads (FPKM) of all annotated transcripts in each data set. Of over 28,000 annotated genes, 15,000 constituted more than 99% of transcript-associated fragments of each sample and were included in the downstream analysis (S8A Fig). We next ranked the genes by expression levels in uninfected HeLa cells (S8B Fig, black diamonds). Relative expression levels were elevated in both p150^KO^ (S8B Fig, green diamonds) and ADAR1^KO^ cells (S8B Fig, orange diamonds). We then assessed the expression levels of our 156 ranked genes, which mostly had FPKM values at intermediate to low levels (Fig 3A). Infection or IFN treatment had little to no effect on the expression levels of these genes (compare lanes 1 with 2–4, 5 with 6–8, and 9 with 10–12). The differences in expression of ADAR1 isoforms also had no significant effect on the expression of these genes (Fig 3B).

The added expression values of the 156 ADAR1-edited transcripts constituted about 1% of the total cellular transcripts (Fig 3C). However, only a fraction of the transcribed RNA will actually enter the cytoplasm, since editing frequently occurs in introns (S4A Fig) and UTRs at positions downstream of the annotated polyadenylation site (Fig 3D, blue dashed line). We noticed that for many of these transcripts, only about 10%–30% had elongated UTRs containing the editing sites, whereas the majority of transcripts terminated at the annotated polyadenylation site (most strikingly observed in the *VOPP1* transcript). Considering these facts, we estimate that between 0.5% and 1% of cellular transcripts are ADAR1 edited, or about 1,000 to 2,000 mRNA copies per cell [43].

### Not all ADAR1-edited transcripts are candidates for MDA-5 recognition

Two independent analyses of the ADAR1-edited transcripts in HEK-293T cells [34] and of Alu-dependent association of transcripts with MDA-5 in HEK-293T cells [42] were recently published. As in our analyses, lists of transcript targets were generated. We asked how much overlap there is between the three studies. For this, we compared our top 156 ADAR1-edited transcripts with 100 MDA-5-associated transcripts of Ahmad and colleagues [42] and the top 200 hits of Chung and colleagues [34].

Our analyses and those of Chung and colleagues shared 67 transcripts, whereas only 19 of our transcripts were common with those of Ahmad and colleagues (Fig 3E). The overlap between the Chung and Ahmad studies is 33 transcripts. All three studies identified the same 16 transcripts but ranked them differently (S8C Fig and S1 Data). Among these transcripts, only the nucleolar GTPase *GNL3L* was consistently within the top 12 and the X-pro-aminopeptidase *XPNPEP3* consistently within the top 30 (S8C Fig and S1 Data). From these 3-way analyses, we conclude that not all ADAR1-edited transcripts are strong candidates for innate immunity activation through MDA-5 recognition. Which of the transcripts identified here are responsible for PKR and IFN regulatory transcription factor 3 (IRF3) activation remains unclear.

### Virus replication is restricted in ADAR1-deficient cells

MeV infections are best suited to characterize ADAR1 activity because under certain circumstances, the MeV negative-strand RNA genome can tolerate clusters of ADAR-diagnostic transitions [24,25]. This genome, which is tightly encapsidated by nucleoprotein (N) [44], usually does not form duplex RNA structures that can be edited by ADAR1. However, when encapsidation fails, ADAR1 can edit MeV genomes. MeV-C^KO^ is particularly useful to study ADAR1 activity because it generates high amounts of dsRNA-forming DI RNA [22,26] (S9 Fig), activating intrinsic immunity [23,27–29,45]. MeV-C^KO^ retains the ability to block IFN induction as well as IFN signaling pathways through the expression of other viral proteins [46–55]. Although other C protein functions exist [56,57], control of replication accuracy [22,26,58–62] is most important to prevent innate immunity activation.

**Fig 4:**
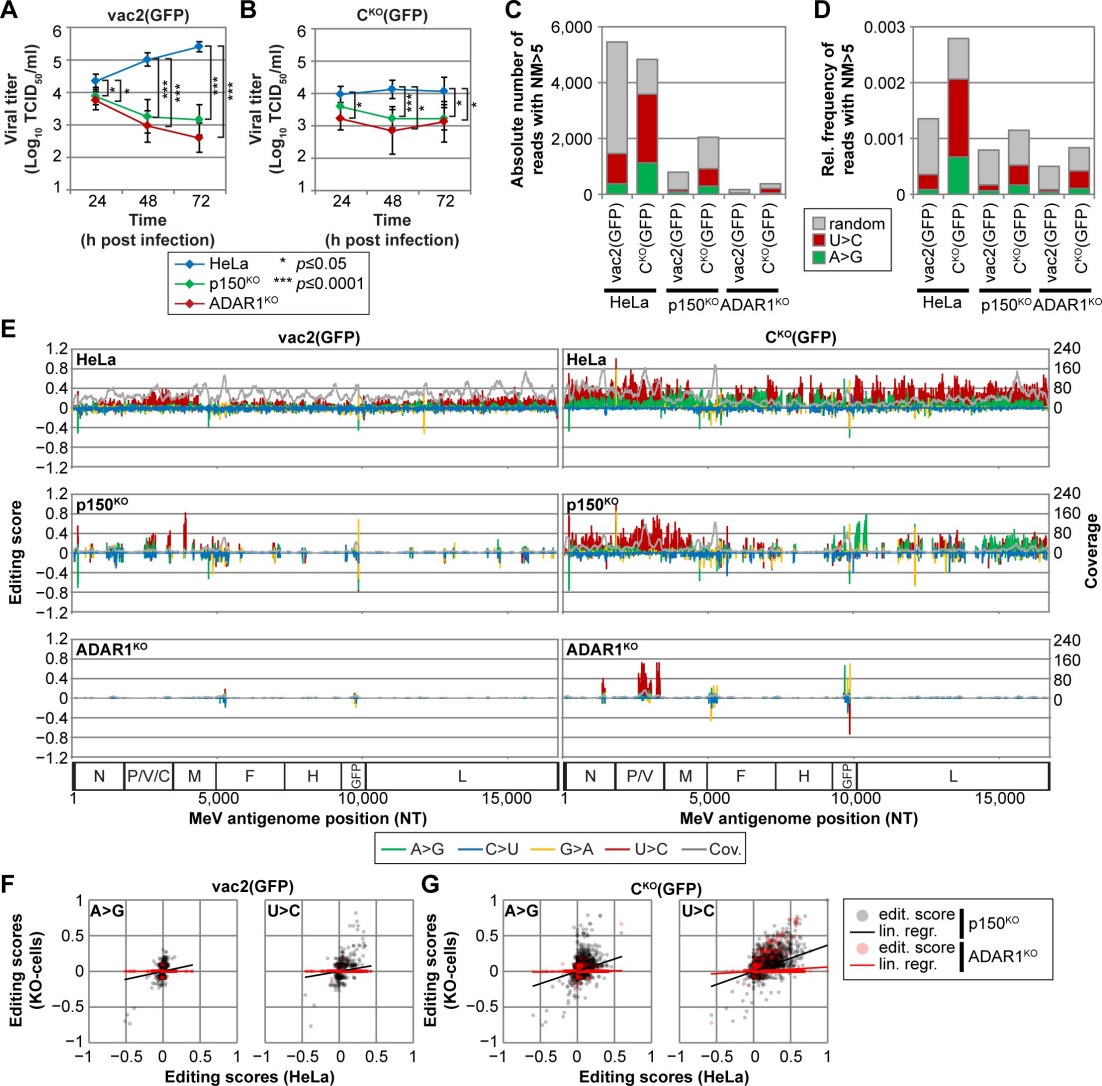
ADAR1 edits MeV genomes and is required for efficient viral replication. (A and B) Growth curve analyses of (A) MeV-vac2(GFP) and (B) MeV-C^KO^(GFP) in HeLa cell lines infected at an MOI of 0.1 and harvested at the time points indicated. Values are average ± standard deviation of *n* = 5 for each time point. For p150^KO^ cells, 3 replicates were generated on clone B13 and 2 replicates on clone C10. For ADAR1^KO^ cells, 3 replicates were generated on clone E7 and 2 replicates on clone E2. Significance was determined by unpaired two-tailed Student’s *t* test and is indicated with asterisks (*, *P* < 0.05; ***, *P* < 0.0001). Underlying values can be found in S1 Data. (C) Absolute number of viral reads with >5 mutations (“NM>5”) in RNAseq samples. Underlying values can be found in S1 Data. (D) Frequency of these reads relative to total number of MeV-specific reads. U>C: reads with predominantly U>C mutations (red); A>G: reads with predominantly A>G mutations (green). Underlying values can be found in S1 Data. (E) Editing scores of MeV-vac2(GFP) and MeV-C^KO^(GFP) genomes from HeLa, p150^KO^, and ADAR1^KO^ infections. Scores are shown for transitions (A>G, green; U>C, red; G>A, orange; C>U, blue) and a read coverage (gray) of at least 10. (F and G) Correlation of (F) MeV-vac2(GFP) and (G) MeV-C^KO^(GFP) genome editing between HeLa and p150^KO^ cells (gray dots and black line) or HeLa and ADAR1^KO^ cells (red dots and line). ADAR1, adenosine deaminase acting on RNA 1; ADAR1^KO^, fully ADAR1-deficient; KO, knock-out; lin. regr., linear regression; MeV, measles virus; MOI, multiplicity of infection; NT, nucleotide; p150^KO^, selectively ADAR1^p150^-deficient; RNAseq, RNA sequencing.

We infected the HeLa-derived cell lines with two reporter viruses, the vaccine-equivalent strain MeV-vac2(GFP) and its isogenic mutant MeV-C^KO^(GFP). During the first 24 h of infection, both viruses replicated to about 10^4^ TCID_50_/ml (Fig 4A and 4B and S1 Data), reaching slightly lower titers in p150^KO^ and ADAR1^KO^ cells compared to HeLa cells. MeV-vac2(GFP) continued to replicate in HeLa cells for the next 48 h, but its replication was completely inhibited in p150^KO^ and ADAR1^KO^ cells at later time points (Fig 4A and S1 Data). MeV-C^KO^(GFP) replication efficiency was similar to that of MeV-vac2(GFP) for the first 24 h but thereafter stopped in all three cell lines (Fig 4B and S1 Data). These observations can be explained as follows. Because of the lack of C protein expression, MeV-C^KO^(GFP) stocks contain large amounts of DI genomes (S9 Fig). These are amplified to high levels already during the initial phase of replication, interfering with the replication of full-length genomes and causing innate immune activation. In contrast, MeV-vac2(GFP) stocks contain minimal amounts of DI genomes (S9 Fig). Even if DI genomes were generated at late MeV-vac2(GFP) infection stages, innate immunity activation may have limited consequences [63]. Thus, ADAR1 deficiency preferentially impacts MeV-C^KO^(GFP) replication.

### ADAR1^p150^ frequently edits defective genomes

We then asked how frequently ADAR1 edits MeV genomes. For this, we amplified both MeV-vac2(GFP) and MeV-C^KO^(GFP) on HeLa and ADAR1-modified cells, purified ribonucleocapsids (RNPs) (S10A Fig), and analyzed them by RNAseq. We obtained purity levels ranging from 92% to 11% (S10B Fig and S1 Data), with coverages of 10^3^ to 10^5^ reads per nucleotide (S10C Fig, gray areas). We extracted reads with at least 5 differences from the reference sequence (S10C Fig, colored areas), which were evenly distributed over the MeV-vac2(GFP) genome but accumulated on either MeV-C^KO^(GFP) genome end, consistent with amplification of DI genomes in these infections [26]. Many of these reads had sudden interruptions of collinearity with the MeV genome, probably reflecting recombination artifacts during library preparation (Fig 4C and 4D, gray color and S1 Data). Reads with predominant A>G or U>C transitions were more abundant after replication in HeLa than in p150^KO^ and ADAR1^KO^ cells, consistent with expectations (Fig 4C and S1 Data). Only about 1 in 3,000 reads of MeV-vac2(GFP) genomes had ADAR1 mutations, whereas 1 in 500 reads of MeV-C^KO^(GFP) genomes were ADAR1 edited (Fig 4D and S1 Data). The 2:1 ratio of U>C- to A>G-mutated reads reflects the ratio of negative-strand to positive-strand MeV genomic RNA in virus preparations [64]. Thus, although coverage of MeV-vac2(GFP) genomes with mutated reads was similarly high as that of MeV-C^KO^(GFP) genomes, U>C and A>G transitions were predominantly introduced into the MeV-C^KO^(GFP) genomes.

We next calculated editing scores for each nucleotide of the two viral genomes amplified in each cell line (Fig 4E). A>G and U>C editing scores in p150^KO^ cells were strongly reduced compared to HeLa cells (Fig 4F and 4G) and nearly absent in ADAR1^KO^ cells. Residual A>G and U>C transitions in ADAR1^KO^ samples may be due to edited genomes and/or DI RNAs in the virus inocula, which were generated in ADAR1-expressing Vero cells (S11A Fig).

The MeV-C^KO^(GFP) genome was more accessible to ADAR1 than the MeV-vac2(GFP) genome. Over 30% of A residues and nearly 60% of U residues in MeV-C^KO^(GFP) showed editing scores of ≥0.05, whereas only 8% of A and 33% of U residues were converted at equal frequencies in MeV-vac2(GFP) (S11B Fig and S1 Data). Neither virus genome accumulated significant C>U or G>A transitions, which could have been indicative for apolipoprotein B mRNA editing enzyme, catalytic polypeptide-like (APOBEC) activity [65]. The nucleotide sequences surrounding the edited sites conferred to the ADAR1-specific pattern previously described [26,66] (S11C and S11D Fig and S1 Data). Altogether, these data document that ADAR1^p150^ is crucial for editing viral genomes and that it more frequently edits genomes of C^KO^ than those of standard MeV.

### Catalytically active ADAR^p150^ counteracts immunity activation and restores virus growth

**Fig 5:**
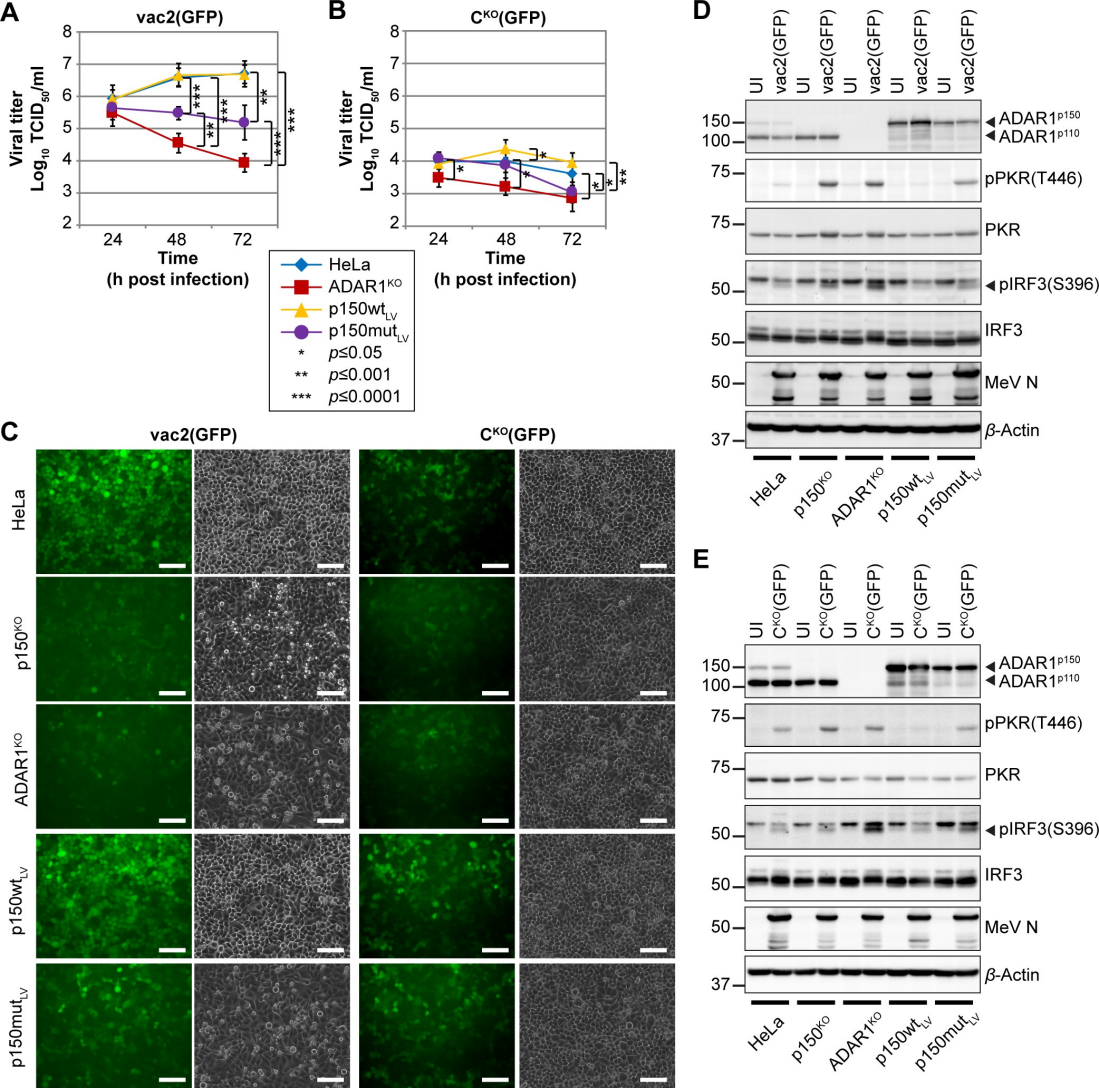
ADAR1^p150^ rescues MeV growth restriction and suppresses intrinsic immunity activation. (A and B) Growth curve analyses of (A) MeV-vac2(GFP) and (B) MeV-C^KO^(GFP) in HeLa, ADAR1^KO^ (clone E7), p150wt_LV_, and p150mut_LV_ cells infected at an MOI of 0.1 and harvested at the time points indicated. Values are average ± standard deviation of *n* = 6 for each time point. *P* values were determined by unpaired, two-tailed Student’s *t* test (*, *P* < 0.05; **, *P* < 0.001; ***, *P* < 0.0001). Underlying values can be found in S1 Data. (C) GFP expression of MeV-vac2(GFP) and MeV-C^KO^(GFP) in ADAR1-modified cells infected at an MOI of 3. Images were taken at 24 h post infection and show GFP fluorescence (green signal) and corresponding phase contrast. Scale bar equals 100 μm. (D and E) Western blot analyses of MeV-vac2(GFP) (D) and MeV-C^KO^(GFP) (E) infected cell lysates. Cells were infected at an MOI of 3 or were left UI and harvested 24 h post infection. ADAR1, adenosine deaminase acting on RNA 1; ADAR1^KO^, fully ADAR1-deficient; GFP, green fluorescent protein; IRF3, interferon regulatory transcription factor 3; MeV, measles virus; MOI, multiplicity of infection; N, nucleoprotein; p150^KO^, selectively ADAR1^p150^-deficient; p150mut_LV_, catalytically inactive ADAR1^p150^; p150wt_LV_, wild-type ADAR1^p150^; pIRF3, phospho-IRF3; PKR, protein kinase R; pPKR, phospho-PKR; UI, uninfected.

To further characterize the role of ADAR1^p150^ in the antiviral response, we complemented ADAR1^KO^ cells with either a catalytically active (p150wt) or inactive (p150mut) cytoplasmic isoform and assessed whether these proteins rescue MeV replication. As shown in Fig 5A and 5B, MeV-vac2(GFP) and MeV-C^KO^(GFP) growth kinetics were almost identical in HeLa (blue lines) and p150wt_LV_ cells (orange lines) (see also S1 Data). Consistently, green fluorescent protein (GFP) expression in p150wt_LV_ cells was at levels similar to those of standard HeLa cells (Fig 5C), but MeV-C^KO^(GFP) replication was still restricted in p150wt_LV_ cells. Expression of the catalytically defective mutant resulted in intermediate complementation levels, as measured by growth kinetics (Fig 5A and 5B, purple lines) and GFP expression analyses (Fig 5C).

Since this experiment suggests that p150mut_LV_ could exert its proviral activity just by binding to dsRNA, we characterized the antiviral response in the five relevant cell lines. Whereas MeV-vac2(GFP) infection induces minimal levels of PKR and IRF3 phosphorylation in standard HeLa cells (Fig 5D), both antiviral pathways are strongly activated upon infection in p150^KO^ and ADAR1^KO^ cells. Moreover, standard ADAR1^p150^ fully suppresses this activation, whereas mutant ADAR1^p150^ only partially suppresses PKR and IRF3 phosphorylation. We observed a similar effect of the expression of different ADAR1 isoforms and mutants on the antiviral response to MeV-C^KO^(GFP) (Fig 5E). Thus, ADAR1 dsRNA binding and catalytic activity are both required to suppress PKR activation.

**Fig 6:**
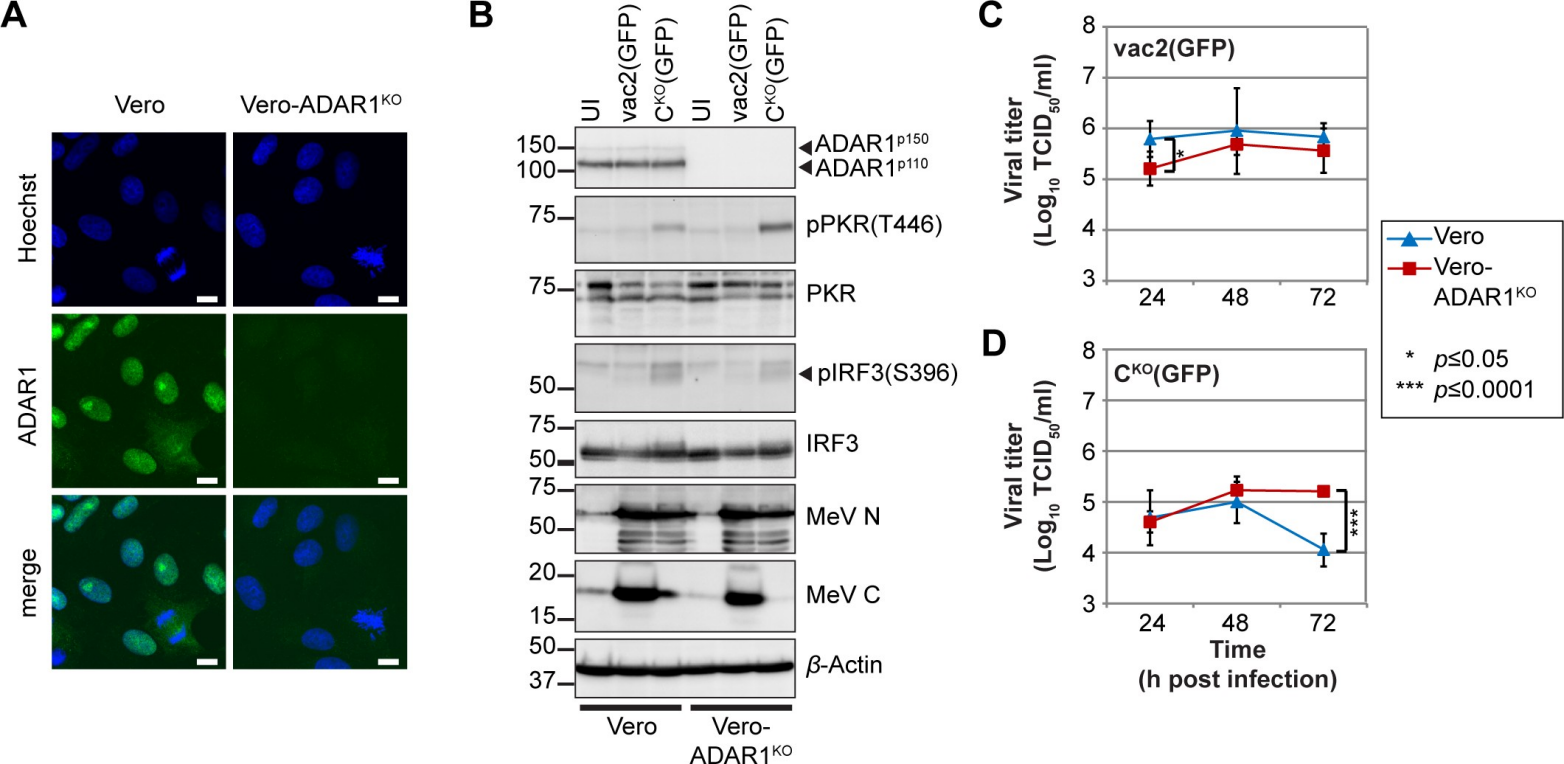
ADAR1 only has a minor effect on MeV replication in IFN-incompetent Vero cells. (A) Confocal immunofluorescence staining of Vero and Vero-ADAR1^KO^ cells. Nuclear staining (Hoechst) in blue, ADAR1-specific staining in green. Scale bar equals 10 μm. (B) Western blot analysis of Vero and Vero-ADAR1^KO^ cell lysates UI or infected with MeV-vac2(GFP) or MeV-C^KO^(GFP) at an MOI of 0.1, 32 h post infection. (C and D) Growth curve analyses of (C) MeV-vac2(GFP) and (D) MeV-C^KO^(GFP) in Vero and Vero-ADAR1^KO^ cells infected at an MOI of 0.1 and harvested at indicated time points. Values are average ± standard deviation of *n* = 6 for each time point. *P* values were determined by unpaired, two-tailed Student’s *t* test (*, *P* ≤ 0.05; ***, *P* ≤ 0.0001). Underlying values can be found in S1 Data. ADAR1, adenosine deaminase acting on RNA 1; ADAR1^KO^, fully ADAR1-deficient; IFN, interferon; IRF3, IFN regulatory transcription factor 3; MeV, measles virus; MOI, multiplicity of infection; N, nucleoprotein; pIRF3, phospho-IRF3; PKR, protein kinase R; pPKR, phospho-PKR; UI, uninfected.

To assess whether activation of intrinsic immunity is directly responsible for virus growth inhibition, we knocked out ADAR1 in IFN-incompetent Vero cells [67] (Fig 6A). If so, replication of MeV-vac2(GFP) and MeV-C^KO^(GFP) is expected to reach similar levels in both Vero and Vero-ADAR1^KO^ cells. Indeed, this is the case, as monitored by GFP expression (S12 Fig) and western blot analyses of viral N and C protein expression (Fig 6B). PKR and IRF3 activation were similar in Vero-ADAR1^KO^ and parental Vero cells (Fig 6B). Accordingly, MeV-vac2(GFP) replication was not significantly reduced in Vero-ADAR1^KO^ cells compared to Vero cells (Fig 6C and S1 Data). Growth of MeV-C^KO^(GFP) in Vero-ADAR1^KO^ cells was even slightly enhanced (Fig 6D and S1 Data). Thus, MeV replication depends on the immune-regulatory effect of ADAR1^p150^ editing of endogenous and viral dsRNA.

### The PKR-mediated stress response controls MeV infection

To determine whether MeV infection of ADAR1^KO^ cells is controlled by the RLR-mediated IFN response, the PKR-mediated cellular stress response, or partially by either pathway, we sought to inactivate these responses. Towards this end, we generated ADAR1^KO^-MAVS^KO^ and ADAR1^KO^-PKR^KO^ cells (S13 Fig). We designed a clustered regularly interspaced short palindromic repeat (CRISPR)/CRISPR-associated 9 (Cas9) approach targeting functional full-length MAVS (FL-MAVS) (S13A and S13C Fig) but not ΔMAVS lacking the essential caspase activation and recruitment domain (CARD) [68]. Similarly, we inactivated PKR through CRISPR/Cas9 targeting of parts of its gene encoding the RBMs (S13B and S13C Fig). As expected, ablation of FL-MAVS expression affected phosphorylation of IRF3 upon transfection of poly(I:C), whereas it did not affect phosphorylation of PKR (S13C and S13D Fig and S1 Data). Vice versa, deletion of PKR had no impact on IRF3 activation (S13C and S13D Fig and S1 Data).

In single-cycle infections of 5 independent clones, GFP expression of either MeV-vac2(GFP) or of MeV-C^KO^(GFP) in ADAR1^KO^-MAVS^KO^ cells (S14C and S14G Fig) was similar to that in ADAR1^KO^ cells (S14B and S14E Fig). In contrast, GFP expression levels of both viruses in 5 independent clones of ADAR1^KO^-PKR^KO^ cells (S14D and S14H Fig) reached similar levels as in HeLa cells (S14A and S14E Fig). Thus, the proviral role of ADAR1 in HeLa cells is mostly mediated through suppression of PKR-mediated stress responses.

**Fig 7:**
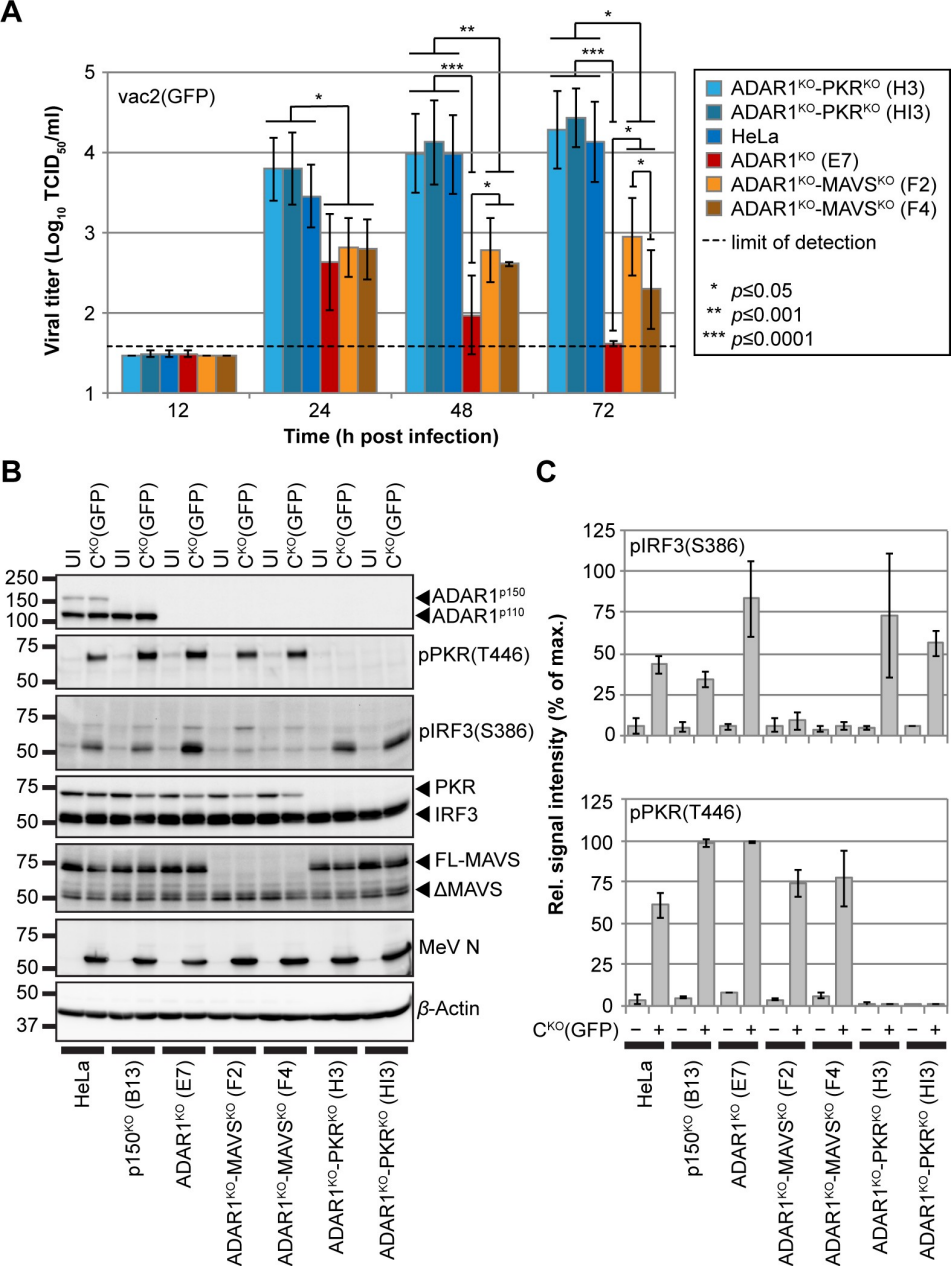
PKR activation elicits a stronger antiviral response than MAVS-mediated IFN induction. (A) Growth curve analyses of MeV-vac2(GFP) in HeLa, ADAR1^KO^ (clone E7), ADAR1^KO^-MAVS^KO^ (clones F2 and F4), and ADAR1^KO^-PKR^KO^ cells (clones H3 and HI3) infected at an MOI of 0.1 and harvested at indicated time points. Values are average ± standard deviation of *n* = 6 for each time point. *P* values were determined by unpaired, two-tailed Student’s *t* test (*, *P* ≤ 0.05; **, *P* ≤ 0.001; ***, *P* ≤ 0.0001). Underlying values can be found in S1 Data. (B) Western blot analysis of cell lysates of UI or MeV-C^KO^(GFP)-infected cells at an MOI of 3, 24 h post infection. (C) Quantification of the pIRF3(S386) signals (top panel) and pPKR(T446) signals (bottom panel) shown in (B). Values are average ± standard deviation of two independent experiments. Underlying values can be found in S1 Data. ADAR1, adenosine deaminase acting on RNA 1; ADAR1^KO^, fully ADAR1-deficient; FL-MAVS, full-length MAVS; IFN, interferon; IRF3, IFN regulatory transcription factor 3; MAVS, mitochondrial antiviral signaling protein; MeV, measles virus; MOI, multiplicity of infection; N, nucleoprotein; p150^KO^, selectively ADAR1^p150^-deficient; pIRF3, phospho-IRF3; PKR, protein kinase R; pPKR, phospho-PKR; UI, uninfected.

Multicycle growth curve analyses of MeV-vac2(GFP) with 2 clones of each double-KO cell line confirmed this conclusion (Fig 7A and S1 Data). Virus titers in ADAR1^KO^-PKR^KO^ cells were similar to those of HeLa cells at any time point. In contrast, virus titers in ADAR1^KO^-MAVS^KO^ cells were much lower than those of HeLa cells. At 48 and 72 h post infection, titers in ADAR1^KO^-MAVS^KO^ cells were higher than those in ADAR1^KO^ cells. These data indicate that PKR elicits an immediate antiviral response that is regulated by ADAR1. Moreover, the MAVS-mediated IFN response to MeV infection, which is effective at later stages, is also regulated by ADAR1. Finally, western blot analyses of C^KO^(GFP)-infected cells (Fig 7B) show that IRF3 activation in ADAR1^KO^-PKR^KO^ cells remains strong, whereas it is reduced in ADAR1^KO^-MAVS^KO^ cells (Fig 7C, upper diagram and S1 Data). In contrast, PKR activation is not altered in ADAR1^KO^-MAVS^KO^ cells (Fig 7C, lower diagram and S1 Data), confirming the predominant antiviral effect of PKR in MeV infection.

## Discussion

### Cellular duplex RNAs: Origin and disposal

Consistently with two recent studies [34,42], we report here that one essential function of ADAR1 is to edit duplex RNA structures located in the 3′ UTR of pol II transcripts. These duplex structures are formed by integrated inverted retrotransposable elements, most frequently Alu elements. We characterized more than 150 highly ADAR1-edited structures, whose prevalence accounts for the massive levels of A>I editing in human cells [8,69]. The editing patterns of HeLa cell transcripts are recapitulated not only in data sets from human donors but also in those from macaques, in which A>I editing occurs in Alu-lineage repeats selectively conserved among primates. In addition to these duplex structures in pol II transcripts, similar structures in noncoding transcripts, which are also ADAR1 substrates [5], may contribute to innate immunity activation by increasing the pool of transcripts with duplex RNA.

Reexamination of mouse ADAR1 editing data [14] reveals that a few transcripts are edited based on dsRNA structures conserved across mammalian orders. This was surprising because mice lack primate-specific Alu repeats. Their genomes have accumulated rodent-specific B1 elements instead. However, like Alu elements, B1 elements derive from 7SL RNA [41]. Thus, ADAR1 editing may have originally targeted the same transposon class. On the other hand, it was reported that retro-elements can activate innate immunity and in particular that endogenous retroviruses can trigger IFN induction [70]. Although this mechanism is plausible, we note that inverted retro-elements embedded in pol II transcripts are more prevalent than bona fide endogenous retroviruses.

Although 67 of the 156 ADAR1-edited transcripts from our study are the same as those identified by Chung and colleagues using a similar ADAR1 gene knock-out approach [34], only 19 were the same as those characterized by Ahmad and colleagues as binding MDA-5 [42]. The simplest explanation for this is that ADAR1 edits more transcript than those MDA-5 would recognize. This would be consistent with our observation that the PKR-mediated cellular stress response may operate in infected cells in addition to the RLR-mediated IFN response.

The overlap of our data with those of Chung and colleagues was more extensive but incomplete. Since the two analyses were performed on different cell lines, it is possible that ADAR1 editing activity is cell type dependent. However, different methodologies applied to evaluate gene expression may account for most differences. Our comparison of HeLa cell–derived editing with editing of data sets from human donors supports this assumption. When transcripts were highly expressed in different samples, they exhibited editing patterns nearly identical to those of HeLa cells. However, editing could not be detected in transcripts expressed at low levels in certain samples. This reflects the limited sensitivity of the assay rather than a significant difference.

### Frequency of RNA editing and collateral damage of viral genomes

Here, we took advantage of a vaccine-lineage MeV and an isogenic mutant generating excess DI RNA (C^KO^) to measure the ADAR1 editing frequency. These DI RNAs have the ability to form panhandle dsRNA structures, which are similar to inverted Alu repeat stem-loops that are targeted by ADAR1. The amount of DI RNA generated during standard MeV infection is low but not zero. Consistently, we detected only about 1,500 edited reads in 32,000 genome equivalents—an average of 1 read per 21 genomes. To account for contiguous reads derived from the same editing event, we correct this number to 1 in 20–100 edited genomes. In C^KO^ genomic RNA, the ratio of edited reads was 6 times higher. Since about 1,000 MeV genomes are produced per infected cell [64], in standard infections 10–50 genomes per average cell are edited, whereas in C^KO^ infections, 60–300 genomes per cell are edited. In contrast, more than 1,000 endogenous transcripts per cell are edited. This suggests that even during infection with a defective virus, duplex structures of cellular origin may be more abundant than those of viral origin.

Whereas editing of DI RNA accounts for the proviral activity of ADAR1, editing of regular genomes would have antiviral properties [71] by inactivating essential gene products. However, editing of regular genomes is expected to be inefficient, since these genomes are fully encapsidated. Indeed, we rarely detected ADAR1 editing in parental MeV infections. Whereas this study has focused on editing of immunostimulatory RNA by ADAR1 and the regulation of innate immune responses, it will be worthwhile to investigate the long-term effects of ADAR1 editing activity on viral genome evolution and quasi-species distribution. Our Vero-ADAR1^KO^ cells, which allow efficient MeV replication in the absence of ADAR1, are a valuable tool for this purpose.

### Editing-dependent and editing-independent components of innate immunity activation

Partial recovery of viral replication through overexpression of a catalytically inactive ADAR1 suggests that its immunoregulatory function has editing-dependent and editing-independent components. The editing-independent function may be due to dsRNA-binding competition with immune sensors such as PKR and MDA-5. Template competition is a mechanism of action shared by many dsRNA-binding proteins, including the influenza A virus NS1 [72] and the vaccinia virus E3L [73]. ADAR1^p110^ has editing-independent functions, such as protection of mRNAs from Staufen-1-mediated decay [74].

For prevention of innate immunity activation, however, simply binding to dsRNA seems insufficient. Evidence for this is found in mice, in which a homozygous mutation E861A in ADAR1, disrupting catalytic activity, exhibits the same embryonically lethal phenotype [14] as full ADAR1 knock-out mice [75]. In addition, most ADAR1 mutations associated with AGS6 are found in the deaminase domain, while the RBMs remain functional [19]. Altogether, these observations and our data indicate that only catalytically active ADAR1 has full immunoregulatory and proviral function.

### A model for the regulatory function of ADAR1 in autoimmunity and infection

**Fig 8:**
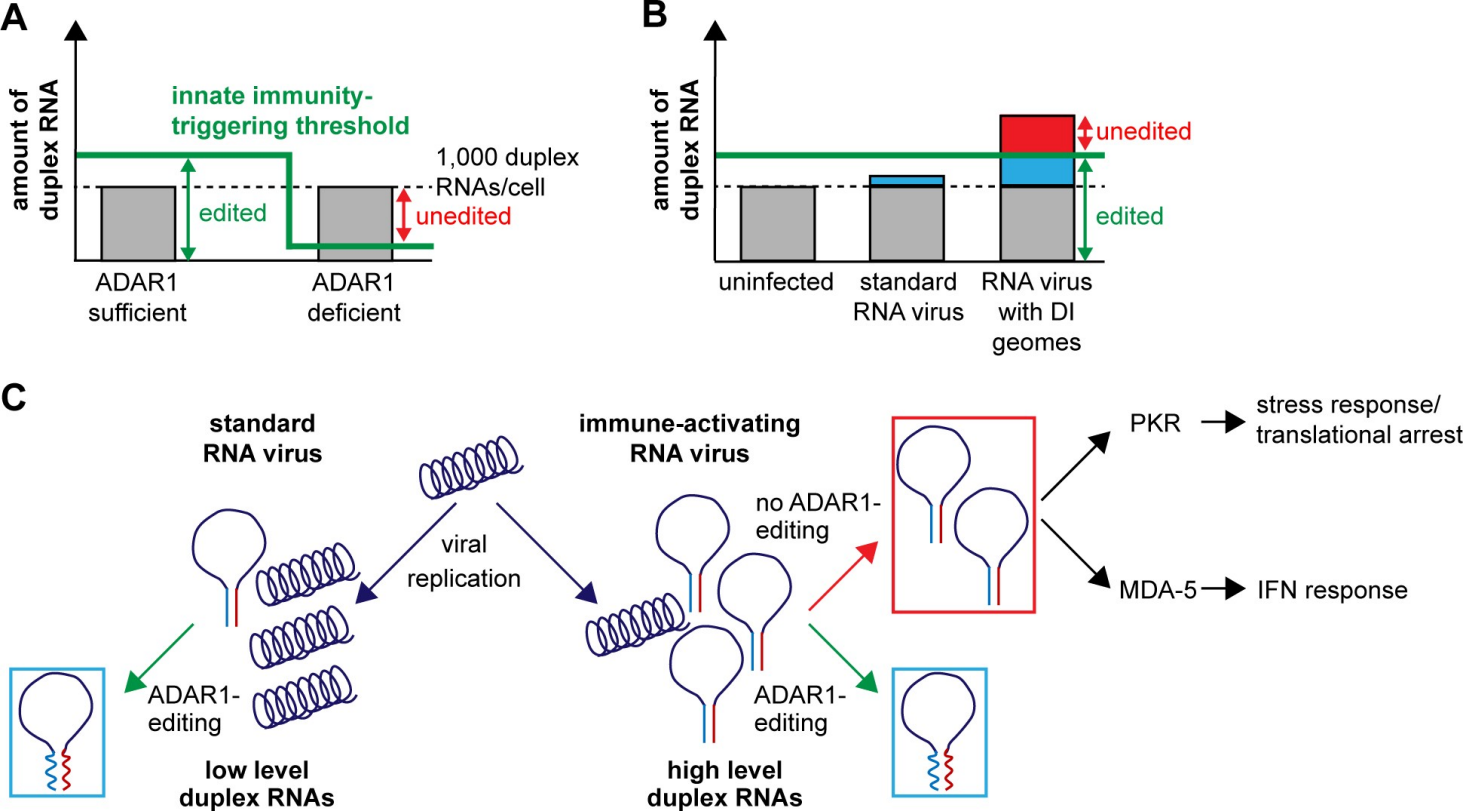
Models for the regulatory function of ADAR1 in autoimmunity and infection. (A) In ADAR1-sufficient cells, transcribed cellular duplex RNA (about 1,000 transcripts under homeostatic conditions, black dashed line) is efficiently edited (green arrow). Thus, no activation of innate immunity occurs. ADAR1 expression level sets the threshold for innate immunity activation (green line). In ADAR1-deficient cells, the threshold is decreased. Levels of transcribed duplex RNA remain equal, but duplexes are not edited and innate immunity is triggered (red arrow). (B) A standard RNA virus (e.g., MeV-vac2) generates low amounts of dsRNA (blue), which is efficiently edited by ADAR1 and thus insufficient to activate innate immunity. In contrast, an RNA virus with DI genomes (e.g., MeV-C^KO^) generates large amounts of duplex RNA (blue and red). ADAR1 still edits some of it (blue), but unedited dsRNA activates innate immunity (red). (C) Schematic representation of the generation of immunogenic duplex RNA (panhandle structures) during viral infection and the impact of ADAR1 on PKR- and MDA-5-mediated innate immunity activation by these RNAs. ADAR1, adenosine deaminase acting on RNA 1; DI, defective interfering; dsRNA, double-stranded RNA; IFN, interferon; MDA-5, melanoma differentiation–associated gene 5; MeV-C^KO^, MeV unable to express C protein; PKR, protein kinase R.

In Fig 8, we present a model of the ADAR1^p150^ mechanism of action consistent with our data, along with evidence provided by multiple studies in mice [2,13–16,75–77], human cells [17,30,34], and human participants [19]. In normal cells, ADAR1 recognizes and alters dsRNA structures in constitutively expressed transcripts and thus prevents autoimmune activation of PKR and MDA-5 (Fig 8A, ADAR1 sufficient). Thus, ADAR1 activity allows the cell to tolerate a certain amount of endogenous duplex RNA, setting a threshold for immune activation. This threshold may vary with the expression levels of ADAR1 in different cell types. In an environment in which ADAR1 is missing or lacks catalytic activity, the threshold is very low (Fig 8A, ADAR1 deficient). Unedited transcripts are recognized by innate immune receptors and induce an autoimmune response. This prevents efficient replication of viruses. Indeed, a standard MeV generating small amounts of dsRNA replicates less efficiently in p150^KO^ or ADAR1^KO^ cells than in parental HeLa cells. Reduced replication of standard MeV in ADAR1-deficient cells can be monitored already 24 h post infection and becomes more pronounced at later infection stages. Thus, MeV replication is slowed down from the beginning and completely inhibited eventually.

Our model also accounts for the immune-activating properties of the C^KO^ virus (Fig 8B and 8C). This virus generates DI RNA from the onset of replication, which adds on top of cellular dsRNA transcripts, at some point exceeding the threshold of efficient ADAR1 editing (Fig 8B, right column). Viral DI RNA, partially edited or unedited, then triggers innate immunity. The width of the gap between the amount of cellular duplex RNA and activation threshold determines how much viral dsRNA can be tolerated by cells before innate immunity activation occurs. For most-effective pathogen detection, the gap should be narrow. Innate immunity activation can occur by more than one mechanism: we observed parallel activation of the PKR-mediated cellular stress response and RLR-mediated IFN induction in ADAR1-deficient cells.

In summary, ADAR1 sets a threshold for intrinsic immunity activation by cellular or viral duplex RNA. By adjusting the intrinsic immune activation threshold and protecting cells from translational shutdown, ADAR1^p150^ provides cover for viruses, which take advantage of enhanced tolerance to duplex RNA accidentally generated during their replication.

## Methods

### Cell lines and media

HEK293T/17 (Cat. #CRL-11268; ATCC, Manassas, VA, United States), HeLa (Cat. #CCL-2; ATCC), and HeLa cell derivatives were kept in DMEM (Cat. #SH30022.01; GE Healthcare Life Sciences, Pittsburg, PA, United States) supplemented with 10% fetal bovine serum (FBS; Cat. #10437–028; Thermo Fisher Scientific) and 1x Penicillin/Streptomycin (Pen/Strep; Cat. #30-002-CI; Corning, Corning, NY, United States). For selection of lentivirus-transduced HeLa cells, puromycin (Cat #P7255-100MG; Sigma Aldrich, St. Louis, MO, United States) was added to the growth media at a final concentration of 1 μg/ml. Vero (Cat. #CCL-81, ATCC) and Vero-ADAR1^KO^ cells were cultivated in DMEM supplemented with 5% FBS and 1x Pen/Strep.

### Generation of ADAR1 knock-out cell lines

HeLa p150^KO^ and ADAR1^KO^ cell lines were generated by CRISPR/Cas9-nickase (Cas9n). For this, pairs of Cas9n cleavage sites in Exon 2 were identified using the ATUM online tool (https://www.atum.bio/eCommerce/cas9/input; ATUM, Newark, CA, United States). gRNAs upstream of M296 (ADAR1^p110^ start codon) were selected for ADAR1^p150^-specific knock-out, and gRNAs downstream of M296 were selected for general ADAR1 knock-out. gRNAs (p150^KO^ site B: B-minus-top, CACCG GAAACCTTGGCCGGAGTCC; B-minus-bottom, AAAC GGACTCCGGCCAAGGTTTCC C; B-plus-top, CACCG CTACTTGCCTCCAGTACCAG; B-plus-bottom, AAAC CTGGTACTGGAGGCAAGTAG C; p150^KO^ site C: C-minus-top, CACCG GTAGCTTGCCCTTCTTTGCC; C-minus-bottom AAAC GGCAAAGAAGGGCAAGCTAC C; C-plus-top, CACCG AGGCAGGAACACCCCCTTTG; C-plus-bottom, AAAC CAAAGGGGGTGTTCCTGCCT C; ADAR1^KO^ site E: E-minus-top, CACCG ATGATGGCTCGAAACTCACC; E-minus-bottom, AAAC GGTGAGTTTCGAGCCATCAT C; E-plus-top, CACCG ATGCCCTCCTTCTACAGTCA; E-plus-bottom, AAAC TGACTGTAGAAGGAGGGCAT C) were cloned into pSpCas9n(BB)-2A-GFP (Cat. #48140; Addgene, Cambridge, MA, United States) as described [78]. HeLa cells seeded in 6 wells (about 1 × 10^6^ cells/well) were transfected with pairs of 2 plasmids (2 μg of each plasmid) using FuGENE HD (Cat. #E2311; Promega, Madison, WI, United States). Single GFP-positive cells were sorted by flow cytometry and seeded into 96 wells 48 h post transfection. Multiple clones were grown and screened for ADAR1 expression by western blot analysis. Disruption of the ADAR1 open reading frame was confirmed by RNAseq analysis for clones B13 (p150^KO^) and E7 (ADAR1^KO^). Stocks of ADAR1-modified cell clones were amplified and frozen in liquid nitrogen. Vero-ADAR1^KO^ cells were generated accordingly.

### Generation of lentivirus-transduced cell populations

HeLa ADAR1^KO^ cells (clone E7) seeded in 6-well plates were transduced with 100 μl puromycin-selectable lentiviral vector stocks expressing either wild-type ADAR^150^ (p150wt_LV_) or catalytically inactive ADAR1^p150^(H910Q/E912A) (p150mut_LV_). Media were replaced 24 h post transduction with fresh DMEM with FBS and Pen/Strep. Cells were expanded into 60-mm dishes 72 h post transduction, and puromycin was added at a final concentration of 1 μg/ml at this time point. Three days later, cells were again trypsinized (Cat. #25-053-CI, Corning) and seeded into T75 flasks. Expression of ADAR1^p150^ was confirmed by western blot analysis. Frozen cell stocks were generated as mixed populations with heterogenous ADAR1^p150^ expression levels and kept in liquid nitrogen. Puromycin was applied to each cell passage and omitted only prior to experiments.

### Virus strains

Recombinant vaccine lineage MeV-vac2(GFP) and MeV-C^KO^(GFP) expressing enhanced GFP from an additional transcription unit were described previously [26]. Generation of recombinant viruses, stock production, and titration were described previously [79]. Infections were carried out as follows: Cells were seeded 16 to 24 h prior to infection at 50% confluency. Cells were incubated with virus inoculums at indicated multiplicities of infection (MOIs) in low volumes of Opti-MEM (Cat. #31985070; Thermo Fisher Scientific, Waltham, MA, US) for 2 h at 37 °C, after which the inoculums were replaced with fresh DMEM with FBS and Pen/Strep. Cells were processed for downstream analyses at the indicated time points post infection.

### Lentiviral vectors

Constructs for puromycin-selectable lentiviral vectors expressing wild-type or catalytically inactive ADAR1^p150^ were generated as follows: ADAR1^p150^ open reading frames were PCR amplified from plasmids pCDNA6-ADAR1^p150^ and pCDNA6-ADAR1^p150^(H910Q/E912A), respectively [30], using primers (ADAR1-fwd-ClaI, ATATAT AAGCTT ATCGAT GCCACC ATGAAT CCGCGG CAGGGG TAT; ADAR1-rev-XhoI, ATATAT CTCGAG CTATAC TGGGCA GAGATA AAAGTT CTTTTC CTCCT) and cloned into pTsin-IRES-puro [80] using *Cla*I and *Xho*I restriction sites. The resulting constructs (4 μg), along with packaging plasmid (pCMV8.9, 4 μg), envelope plasmid (pVSV-G, 1.3 μg) [80], and a GFP reporter (pCR3-GFP, 0.7 μg), were transfected into HEK-293T/17 cells using 40 μg PEI in 250 μl Opti-MEM [80]. For this, cells were seeded in 100-mm dishes at 50% confluency 16–24 h prior to transfection. Cells were incubated with the transfection mix for 6 h at 37 °C, after which the media was replaced with 10 ml fresh DMEM with FBS and Pen/Strep. Lentiviral vector-containing supernatants were collected 48 h post transfection, sterile filtered through a 0.45-μm-pore filter, aliquoted, and stored at −80 °C.

### IRB and IACUC statement

No human subjects or animals were directly involved in this study. Primary RNAseq data sets of human subjects, macaques, and mice were derived from the GEO database.

### Cell growth and viability assay

Cells grown to confluency in T25 flasks were washed once with PBS and incubated with Versene (Cat. #15040066; Thermo Fisher Scientific) at 37 °C until they started detaching from the surface. Cells were suspended in 10 ml PBS and counted using a Neubauer chamber. A total of 3 × 10^6^ cells were stained with CellTrace Violet (Cat. #C34571; Thermo Fisher Scientific) according to the manufacturer’s instructions. Then, 3 × 10^5^ stained cells were seeded into several 6-well plates with 2 ml growth medium and incubated for the indicated amounts of time (0, 24, 48, 72, 96 h). At each time point, cells were washed once with PBS, detached with Versene, and counted as described above. A total of 1 × 10^5^ cells were diluted in 100 μl 1x binding buffer and stained with FITC-conjugated anti-Annexin V as well as propidium iodide (PI), as described in the manual of the Apoptosis detection kit I (Cat. #BDB556547; BD Biosciences, San Jose, CA, US). After the incubation time, cells were diluted to 500 μl with 1x binding buffer and immediately analyzed by flow cytometry using a LSR II flow cytometer (BD) as well as FlowJo software (v.10) and Microsoft Excel. Briefly, unstained and single-stained control cell populations were used to determine background fluorescence and to compensate cross-fluorescent signals. Triple-stained cells were then analyzed until 30,000 events were collected. For analysis, signals were first gated for single cells using FSC and SSC, as well as FSC-A and FSC-H. Single cells were then subdivided into living, apoptotic, and dead cells according to their Annexin V/PI staining. For each subgroup at each time point, CellTrace Violet fluorescence was then analyzed. Gates for 0–4 divisions were created based on the living HeLa cell population and subjected to the other cell lines and time points.

### Virus growth curve analysis

Cells were seeded in 6-well plates at 50% confluency 16–24 h prior to infection. Infections were carried out at an MOI of 0.1. At indicated time points, supernatants were removed and cells were scraped into 100 μl Opti-MEM per well, followed by 3 freeze/thaw cycles (liquid nitrogen/37 °C). Cell debris was removed by centrifugation (4,000*g*, 4 °C, 10 min). Viral titers of cleared lysates were determined by infecting monolayers of Vero-hSLAM cells [81] with 10-fold dilutions of lysates for 96 h, as described previously [79]. Viral titers were determined using the TCID_50_ method.

### Western blot analysis

Preparation of cytoplasmic extracts, SDS-PAGE and western blot analyses were performed as previously described [22]. Whole-cell lysates were generated by lysing cells of confluent 6-well plates directly in 200 μl denaturing lysis buffer (62.5 mM Tris [pH 6.8]; 2% [w/v] SDS; 10% [v/v] Glycerol; 6 M Urea; 5% [v/v] beta-mercaptoethanol; 0.01% [w/v] Bromophenol blue). Lysates were sonicated (50% output, 2 × 2-s pulses) and stored at −20 °C. For nucleocytoplasmic fractionation, cells were lysed in 100 μl native lysis buffer (10 mM HEPES [pH 7.9]; 200 mM NaCl; 5 mM KCl; 10% [v/v] Glycerol; 0.5% [v/v] NP-40; 1 mM EDTA; 1 mM Na-orthovanadate; 5 mM NaF; 1 mM PMSF; 1 mM DTT; 1% [v/v] Protease inhibitor cocktail [Cat. #P8340-5ML; Sigma Aldrich]; 1% [v/v] Phosphatase inhibitor cocktail 3 [Cat. #P0044-5ML; Sigma Aldrich]) and incubated on ice for 15 min. Nuclei were pelleted by centrifugation at 20,000*g*, 4 °C, 15 min. Supernatants were collected, mixed with equal volumes of 2x Urea sample buffer (200 mM Tris–HCl [pH 6.8]; 8 M Urea; 5% [w/v] SDS; 0.1 mM EDTA; 0.03% [w/v] Bromophenol blue; 1.5% [w/v] DTT), and stored at −20 °C. Nuclei were washed twice with PBS and lysed in 200 μl denaturing lysis buffer. Lysates were stored at −20 °C. Antibodies used for detection were rabbit polyclonal anti-MeV-N_505_ [29]; rabbit polyclonal anti-MeV-C_2_ [29]; rabbit monoclonal anti-ADAR1 (D7E2M; Cat. #14175S; Cell Signaling Technology, Danvers, MA, US); mouse monoclonal anti-ADAR2 (clone 1.3.1; Cat. #MABE889; Millipore Sigma, Burlington, MA, US); rabbit monoclonal anti-PKR (D7F7; Cat. #12297S; Cell Signaling Technology); rabbit polyclonal anti-PKR (K-17; Cat. #sc-707; Santa Cruz Biotechnology, Dallas, TX, US); rabbit monoclonal anti-phospho-PKR(T446) (Cat. #ab32036-100UL; Abcam, Cambridge, United Kingdom); rabbit monoclonal anti-IRF3 (D6I4C; Cat. #11904S; Cell Signaling Technology); rabbit monoclonal anti-phospho-IRF3(S396) (Cat. #4947S; Cell Signaling Technology); rabbit monoclonal anti-phospho-IRF3(S386) (EPR2346; Cat. #AB76493-100UL; Abcam); mouse monoclonal anti-β-Actin, HRP-conjugated (Cat. #A3854-200UL; Sigma Aldrich); goat anti-mouse IgG(H+L), HRP-conjugated (Cat. #401215; Millipore Sigma); and goat anti-rabbit IgG(H+L), HRP-conjugated (Cat. #111-035-144; Jackson Immunoresearch, West Grove, PA, US). Membranes were scanned using a ChemiDoc Imaging System (Biorad, Hercules, CA, US), analyzed, and quantified using the Image Lab software (v 6.0.0 build 25, Biorad).

### IFN treatment

Cells were seeded in 6-well plates at 50% confluency 16–24 h prior to treatment. IFN A/D (Cat. #11200–1; PBL Assay Science, Piscataway, NJ, US) was diluted in fresh DMEM with FBS and Pen/Strep at 1,000 U/ml and added to the cells for 24 h.

### Immunofluorescence staining and confocal microscopy

Approximately 1 × 10^4^ cells/well were seeded in 8-chamber microscopy slides (Cat. #154534; Thermo Fisher Scientific) and fixed with 4% (w/v) paraformaldehyde solution in PBS 24 h later. Cells were then permeabilized with 0.5% (v/v) Triton X-100 in PBS for 5 min, followed by blocking with 2.5% (w/v) bovine serum albumin (Cat. #85040C; Sigma Aldrich) and 1% (w/v) normal goat serum (Cat. #50062Z; Thermo Fisher Scientific) in PBS for 30 min. Cells were then stained with rabbit monoclonal anti-ADAR1 (D7E2M; Cat. #14175S; Cell Signaling Technology) diluted at 1:200 in blocking solution with 0.1% (v/v) Triton X-100 for 2 h and counterstained with AlexaFluor 488-conjugated goat anti-rabbit(H+L) secondary antibody (Cat. #A11008; Thermo Fisher Scientific) for 2 h. Nuclei were stained for 5 min with Hoechst 33258 bis-Benzimide (Cat. #B2883-25MG; Sigma Aldrich) diluted at 1:1,000 in H_2_O and mounted with cover slips and ProLong Gold Antifade Mountant (Cat. #P36930; Thermo Fisher Scientific). Images were taken with a LSM 780 system (Carl Zeiss Microscopy, Thornwood, NY, US) and ZEN 2.1 software (black edition v. 11.0; Carl Zeiss Microscopy).

### RNP purification and RNA extraction

HeLa cells were seeded in 150-mm dishes at 5 × 10^6^ cells per dish 24 h prior to infection, and infections were carried out at an MOI of 0.1. For a typical RNP preparation, 10 dishes were infected and cells were harvested 72 h post infection. Cells were scraped into 5 ml PBS per dish and pelleted in 50-ml conical tubes by centrifugation at 300*g* and 4 °C for 10 min. Cell pellets were resuspended in 3 ml ice-cold Hypotonic buffer (10 mM HEPES [pH 7.5]; 10 mM NaCl; 1.5 mM MgCl_2_) supplemented with 0.65% (v/v) NP-40 substitute and protease inhibitor cocktail (Cat. #11836153001; Roche, Basel, Switzerland) for 30 min on ice. Cell debris was pelleted by centrifugation at 4,000*g* and 4 °C for 15 min. The supernatant was then supplemented with 1% (w/v) sodium deoxycholate and 10 mM EDTA and spun a second time at 20,000*g* and 4 °C for 15 min. The lysate was loaded on top of a discontinuous CsCl gradient in SW41 polypropylene centrifuge tubes (Beckman Coulter, Brea, CA, US). CsCl solutions of different concentrations were prepared in gradient buffer (25 mM Tris [pH 7.5]; 50 mM NaCl; 2 mM EDTA; 0.2% (w/v) sodium lauroyl sarkosinate [sarkosyl]). The discontinuous gradient was prepared as follows (from bottom to top): 1 ml of 40% (w/v) CsCl; 2.5 ml of 30% (w/v) CsCl; 1.5 ml of 25% (w/v) CsCl; 1 ml of 5% (w/v) sucrose. Ultracentrifugation was carried out in a SW41 rotor in a LE-80 ultracentrifuge (Beckman Coulter, Brea, CA, US) at 36,000 rpm and 4 °C for 22 h. RNPs banded about 2 cm above the bottom of the tube and were harvested in approximately 1 ml volume by needle aspiration using a 16-gauge needle and syringe. RNPs were diluted in 8 ml LEH buffer (10 mM HEPES [pH 7.5]; 100 mM LiCl; 1 mM EDTA), layered over 2 ml of 15% (w/v) sucrose in LEH buffer in SW41 centrifuge tubes and centrifuged a second time at 36,000 rpm and 4 °C for 6 h. Afterwards, the supernatant was discarded, and RNP pellets were carefully resuspended in 1 ml LEH buffer supplemented with 1% (w/v) SDS. Total RNA was extracted from this solution using Trizol LS (Cat. #10296010; Thermo Fisher Scientific) and precipitated with isopropanol according to the manufacturer’s instructions. Precipitated RNA was resuspended in 25 μl nuclease-free H_2_O and stored at −80 °C.

### Northern blot analysis

Ribonuleocapsid-derived RNA or total RNA (5 μl) from an amount of viral stock equivalent to 1 × 10^6^ TCID_50_ was separated on 1% (w/v) denaturing agarose gels supplemented with 2% (v/v) formaldehyde, and northern blot analysis using the DIG-system (Cat. #12039672910; Roche) was performed as described previously [26].

### RNAseq library preparation and Illumina sequencing of viral RNPs

RNP RNA (5 μl) was fragmented for 7.5 min using the Ambion Fragmentation Reagent (Cat. #AM8740; Thermo Fisher Scientific) according to the manual. The samples were then diluted with nuclease-free H_2_O to a final volume of 100 μl, mixed with an equal volume of buffered phenol/chloroform/isoamyl alcohol (Cat. #15593031; Thermo Fisher Scientific) and phase-separated by centrifugation (12,000*g*, 4 °C, 10 min). RNA was precipitated from the aqueous phase using sodium acetate/ethanol overnight at −20 °C followed by centrifugation (20,000*g*, 4 °C, 30 min). Pellets were washed with 70% (w/v) ethanol, dried, and resuspended in 11 μl nuclease-free H_2_O. RNA libraries were prepared using 220–500 ng of total RNA according to the manufacturer’s instructions for the TruSeq Stranded Total RNA Sample Prep Kit (Cat. #RS-122-2201; Illumina, San Diego, CA, US). The concentration and size distribution of the completed libraries was determined using an Agilent Bioanalyzer DNA 1000 chip (Cat. #5067–1504; Agilent, Santa Clara, CA, US) and Qubit fluorometry (Invitrogen, Carlsbad, CA). Libraries were sequenced at 8–14 million reads per sample following Illumina’s standard protocol. The flow cells were sequenced as 300 × 2 paired-end reads on an Illumina MiSeq using MiSeq v2 sequencing kit (Cat. #MS-102-2002; Illumina) and MCS v2.6.2.1 collection software. Base-calling was performed using Illumina’s RTA version 1.18.54.

### RNAseq analysis of RNP RNA

Analysis was performed using the Galaxy platform (https://usegalaxy.org) [82]. Raw BAM files were converted to FASTQ files (SAM-to-FASTQ v. 1.56.1), generating 2 FASTQ files for each data set (split by read group). Adapter sequences were clipped (FASTX Toolkit), using Illumina adapter recognition sequences GATCGGAAGA GCACACGTCT GAACT (read 1 files) or AGATCGGAAG AGCGTCGTGT AGGGA (read 2 files), quality trimmed (3′ ends, sliding window 1, step size 1, quality score ≥20) and quality filtered (35–301 nt length; discard reads with N; >95% of nucleotides with quality scores >30). The resulting FASTQ files were evaluated using FASTQC and then aligned against a custom built genome containing sequences of hg38 (GRCh38.p7, GCA_000001405.25), hsa-45S-pre-rRNA (GenBank NR_046235), and MeV-vac2(GFP)H (GenBank MH144178) using Bowtie2 (v. 2.2.6; sensitive end-to-end) [83]. For further analysis, resulting BAM files were filtered for number of mutations per read (NM > 5; NM > 8; NM > 11) using BAMtools filter (v. 2.4.1). Count tables of BAM alignments were generated using IGVTools [84] and analyzed using Microsoft Excel 2010.

### Total cell transcriptome RNAseq library preparation and sequencing

Total RNA of HeLa, p150^KO^, and ADAR1^KO^ cells untreated, treated with 1,000 U/ml IFN A/D for 24 h, or infected with either MeV-vac2(GFP) or MeV-C^KO^(GFP) at an MOI of 3 for 24 h was extracted using Trizol reagent (Cat. #15596026; Thermo Fisher Scientific) and isopropanol precipitated according to the manufacturer’s instructions. Total RNA (1 μg) was digested with 1 U DNAse I (Cat. #18068015; Thermo Fisher Scientific) in a total volume of 10 μl at 25 °C for 15 min. The reaction was stopped by addition of EDTA and heat inactivation at 65 °C for 10 min according to the manufacturer’s instructions. RNA was purified using the RNeasy cleanup kit (Cat. #74204; Qiagen, Hilden, Germany) according to the instructions and eluted with 30 μl H_2_O_DEPC_. RNA libraries were prepared using 100 ng of total RNA according to the manufacturer’s instructions for the TruSeq Stranded Total RNA Sample Prep Kit (Cat. #RS-122-2201; Illumina). The concentration and size distribution of the completed libraries was determined using an Agilent Bioanalyzer DNA 1000 chip (Cat. #5067–1504; Agilent) and Qubit fluorometry (Thermo Fisher Scientific). Libraries were sequenced at 60–110 million reads per sample following Illumina’s standard protocol using the Illumina cBot and HiSeq 3000/4000 PE Cluster Kit (Cat. #PE-410-1001; Illumina). The flow cells were sequenced as 100 × 2 paired-end reads on an Illumina HiSeq 4000 using HiSeq 3000/4000 sequencing kit (Cat. #FC-410-1001 and #FC-410-1002; Illumina) and HCS v 3.3.52 collection software. Base calling was performed using Illumina’s RTA version 2.7.3.

### RNAseq analysis of total cell transcriptomes

Analysis was performed using the Galaxy platform (https://usegalaxy.org). Raw BAM files were converted to FASTQ files (SAM-to-FASTQ v. 1.126.0), generating 2 FASTQ files for each data set (split by read group). Adapter sequences were clipped (FASTX Toolkit), using Illumina adapter recognition sequences GATCGGAAGA GCACACGTCT GAACT (read 1 files) or AGATCGGAAG AGCGTCGTGT AGGGA (read 2 files) and quality trimmed (3′ ends, sliding window 1, step size 1, quality score ≥30). The resulting FASTQ files were evaluated using FASTQC and then aligned as paired-end reads against hg38 (GRCh38.p7, GCA_000001405.25) using Bowtie2 [83] (v 2.2.6.2, sensitive end-to-end). Resulting aligned BAM files were visualized with the Integrated Genome Viewer (IGV, v. 2.3.98[158]). Count tables of regions of interest were generated using IGVTools and analyzed using Microsoft Excel 2010.

### Gene expression quantification of RNAseq data

BAM files with mapped reads were subjected to the Cufflinks suite [85] implemented on the Galaxy platform (https://usegalaxy.org). Briefly, assembled transcripts were generated using Cufflinks, and a final transcriptome assembly was generated from this using Cuffmerge. Mapped reads were quantified on this assembly using Cuffquant, and normalized expression levels were calculated using Cuffnorm. Heatmaps were generated in Microsoft Excel 2010. For calculation of expression levels relative to GAPDH, average and 95% confidence values of the four samples derived from each cell line were calculated.

### RNAseq analysis of primary human, macaque, and mouse data sets

RNAseq data sets were retrieved from the GEO database using Fastq-dump of the SRA Toolkit (v. 2.8.0, NCBI, Bethesda, MD, US). Human donor RNAseq data were from study GSE57353 [38] (data sets SRR1272457, SRR1272459, SRR1272461, SRR1272763, SRR1272465, SRR1272467, and SRR1272469) and GSE99392 (data sets SRR5626959 and SRR5626960). Macaque tissue data were from study GSE42857 [39] (data sets SRR630492, SRR630493, SRR630494, SRR1261481, and SRR1778441), and mouse tissue data were from study GSE58917 [14] (data sets SRR1501185, SRR1501186, SRR1501187, SRR1501188, SRR1501189, and SRR1501190). FASTQ files were uploaded to the Galaxy platform (https://usegalaxy.org) and split into forward and reverse reads. Reads were aligned to reference genomes human hg38 (GRCh38.p7, GCA_000001405.25), macaque mmul8.0.1 (GCA_000772875.2), or mouse mm9 (GRCm38.p6, GCA_000001635.8) using Bowtie2 (v 2.2.6.2, sensitive end-to-end). Resulting aligned BAM files were visualized with the IGV (v. 2.3.98[158]). Count tables of regions of interest were generated using IGVTools and analyzed using Microsoft Excel 2010.

### GIREMI analysis

Single nucleotide variant (SNV) calling was performed using the SAMtools (v. 1.3) and BCFtools (v. 1.3) [86]. The produced SNV list was passed to the GIREMI (v. 0.2.0) [33], which split it into two groups: RNA editing positions and SNPs, dbSNP (build 138) [87] was used for the GIREMI statistical model evaluation. The resulting tables were imported into Microsoft Excel 2010, and numbers of mutations on each chromosome for each data set were counted. Numbers of A>G mutations were also determined for contiguous chromosome segments of 1,000,000 bp, as well as for individual genes. The list of ADAR1-edited genes was generated by comparing the number of editing sites in HeLa cells with the number in ADAR1^KO^ cells and ranked according to the highest differential. The following inclusion criteria were applied sequentially: Genes were included, 1. if the number of editing sites was ≥8 in HeLa cells; 2. if the ratio of detected editing sites #ADAR1^KO^/#HeLa was ≤0.5; 3. if the number of editing sites per 100,000-bp gene length was ≥10; and 4. if #HeLa/(#ADAR1^KO^ + 1) was ≥1.75.

### Editing score analysis

Read base count tables of regions of interest were generated from aligned BAM files using IGVTools (igvtools count--bases–w 1) and imported to Microsoft Excel 2010. Editing scores (*e*[Ts]) for A>G, C>U, G>A and U>C transitions (Ts) were calculated using function (Eq 1):

$$e(Ts)=\frac{n_{Ts}-(n_{Tv1}+n_{Tv2})}{COV},$$ (1)

with *n*_Ts_ being the read counts of the transition nucleotide at the analyzed position, *n*_Tv1_ and *n*_Tv2_ being the read counts of the respective transversion nucleotides, and *COV* being the total read count at the analyzed position (coverage). Negative editing scores occur if more transversions than transitions are reported and indicate either sequencing artifacts or single nucleotide polymorphisms at the analyzed position.

Significance of the ADAR1-specific transitions was tested against transversions at the same nucleotide position using Pearson chi-squared test with one degree of freedom (Eq 2)

$$\chi^{2}=\frac{{\left(O_{i}-E_{i}\right)}^{2}}{E_{i}+1}=\frac{(n_{Ts}-{(n}_{Tv1}+n_{Tv2}{))}^{2}}{n_{Tv1}+n_{Tv2}+1}$$ (2)

and by the Poisson model–based Wilks log-likelihood ratio that is asymptotically distributed as chi-squared with one degree of freedom:

$$P=2∙\left[\frac{n_{Ts}∙\ln\left(n_{Ts}\right)}{\left(n_{Tv1}+n_{Tv2}+1\right)}-n_{Ts}+(n_{Tv1}+n_{Tv2}+1)\right].$$ (3)

A difference between HeLa and ADAR1^KO^-derived cells in enrichment of any interval by editing scores was calculated based on the Pearson and Poisson-Model chi-squared score estimations across the interval positions. Since the square root of a chi-squared 1 d.f. distributed score *s*_*i*_ is normally N(0, 1) distributed, the sum of $\sqrt{s_{i}}$ over the interval positions divided by square root of the interval length *L*:

$$S_{L}=\frac{\sum_{i=1}^{L}(\sqrt{s_{i})}}{\sqrt{L}}$$ (4)

is also distributed normally N(0,1). For the most differentiating interval in positions 55,453,760–55,459,700 of chromosome 7, the Poisson-Model interval score $S_{L}^{H}=8.9$ in the HeLa cells (pval = 0) and $S_{L}^{Ako}=0.81$ (pval = 0.21) for the same interval in the ADAR1^KO^ cells. A difference between HeLa and ADAR1^KO^ cells in the interval enrichment by the RNA-editing Poisson-Model scores can be estimated by the *S*_*diff*_ statistics:

$$S_{diff}=\frac{S_{L}^{H}-S_{L}^{Ako}}{\sqrt{2}}$$ (5)

that is N(0,1) distributed. For this interval of chromosome 7, the differentiation score *S*_*diff*_ = 5.72 with *P* = 5.3E-09.

### RNA secondary structure prediction

Secondary structures of regions of interest were predicted using the RNAfold WebServer (http://rna.tbi.univie.ac.at//cgi-bin/RNAWebSuite/RNAfold.cgi) [88]. Models shown here are minimum free energy (MFE) models.

### Editing site neighboring nucleotide analysis

Analysis was performed as described previously [66]. Briefly, 9-nucleotide sequences around editing sites (A>G and U>C) were extracted for sites with transition frequencies of ≥20% and a coverage of at least 10 reads/nucleotide. For U>C sites, reverse complementary sequences were analyzed. Relative nucleotide frequencies at each position were calculated and normalized to the U-frequency.

### Quantification and statistical analysis

RNAseq analyses were performed as *n* = 1 of each sample. For analysis of ADAR1 editing in viral genomes, forward and reverse reads of the paired-end sequencing data were analyzed separately, and sites were confirmed to be mutated in both reads.

Cellular transcriptome RNAseq was performed as *n* = 1 of each sample. Editing sites were confirmed in GEO-deposited RNAseq data sets.

Where applicable, Student’s *t* tests were performed for analysis of statistical significance using Graphpad (https://www.graphpad.com/quickcalcs). GIREMI-derived mutation ratios in HeLa and ADAR1^KO^ cell chromosomes were analyzed by one-sample *t* test against an expected value of 1. Viral titers were compared by unpaired, two-tailed *t* tests. Growth kinetics in CRISPR/Cas9-modified cell lines were performed in multiple replicates on 2 independent clones each (see figure legends). Lentivirally transduced cells were treated as puromycin-selected mixed-cell populations. Data are represented as mean ± standard deviation. *P* values are shown by asterisks, if significant (*, *P* ≤ 0.05; **, *P* ≤ 0.001; ***, *P* ≤ 0.0001).

## Supporting information

S1 Fig Generation of p150^KO^ and ADAR1^KO^ cells. (A) Organization of the *ADAR* locus on chr1. Transcription from the constitutively active promoter upstream of exon 1B results in translation of the ADAR1^p110^ isoform from an AUG in exon 2 (M296), indicated by arrow. Transcription from the IFN-inducible promoter upstream of exon 1A results in translation from a start codon within exon 1A (M1), indicated by arrow, giving rise to the ADAR1^p150^ isoform. Green and red triangles (B, C, E) indicate locations of gRNA binding sites for CRISPR/Cas9 targeting. B and C lead to disruption only of ADAR1^p150^, whereas E leads to disruption of both isoforms. (B) Genetic characterization of CRISPR/Cas9 disruption of ADAR1^p150^ in clone B13 and of both isoforms in clone E7. Underlined nucleotides indicate gRNA binding sites; bold nucleotides indicate PAMs. Red highlighted nucleotides indicate insertions or deletions (marked by dashes) causing disruption of ADAR1 open reading frames. Altered amino acids are shown in red above each allele. Three alleles were detected in each clone, indicating that HeLa cells have 3 copies of the *ADAR1* locus. (C) Western blot analysis of CRISPR/Cas9-modified HeLa clones deficient for ADAR1^p150^ (p150^KO^) or both isoforms (ADAR1^KO^). Cells were treated with 1,000 U/ml IFN A/D for 24 h or left untreated. Two independent clones for each knock-out are shown. (D) Confocal immunofluorescence staining of HeLa cell clones with altered ADAR1 expression. Nuclear staining (Hoechst) in blue, ADAR1-specific staining in green. Scale bar equals 10 μm. (E) Western blot analysis of total cell extracts (“T”) and cytoplasmic (“C”) and nuclear fractions (“N”) of HeLa, p150^KO^, and ADAR1^KO^ cells. ADAR1, adenosine deaminase acting on RNA 1; ADAR1^KO^, fully ADAR1-deficient; Cas9, CRISPR-associated 9; chr1, human chromosome 1; CRISPR, clustered regularly interspaced short palindromic repeat; gRNA, guide RNA; IFN, interferon; IFN A/D, recombinant type-I IFN-alpha; p150^KO^, selectively ADAR1^p150^-deficient; PAM, protospacer adjacent motif.
(TIF)

S2 Fig Analysis of growth kinetics and viability of ADAR1-modified HeLa cells. (A) Flow cytometry gating strategy for cell viability. Cells were stained with FITC-conjugated anti-Annexin V for detection of apoptotic cells (*x* axis) and PI for detection of dead cells (*y* axis). Single-cell populations were subdivided into live (Annexin V−/PI−), apoptotic (Annexin V+/PI−), and dead cells (Annexin V−/PI+ and Annexin V+/PI+). (B) Quantification of cell viability of HeLa, p150^KO^, and ADAR1^KO^ cells at various times (in hours) after staining with CellTrace Violet. Underlying values can be found in S1 Data. (C) Analysis of cell division of live (left column), apoptotic (center column), and dead cells (right column) at indicated time points post CellTrace Violet staining. HeLa (second row), p150^KO^ (third row), and ADAR1^KO^ cells (bottom row) were analyzed. Histograms show intensities of CellTrace Violet fluorescence (*x* axes) and relative cell numbers (modal *y* axes). Dashed lines indicate gates for 0, 1, 2, 3, and 4 cell divisions based on live HeLa cell signals (second row of panels, left column). (D) Quantification of the percentage of live HeLa (top diagram), p150^KO^ (center diagram), and ADAR1^KO^ cells (bottom diagram) having undergone *n* divisions at each time point. Black dashed lines indicate time points at which 50% of cells have undergone *n* divisions (DT_50_). (E) Extrapolation of DT_50_ values against number of divisions (*n*) for HeLa (blue), p150^KO^ (green), and ADAR1^KO^ cells (red). The coefficients of the corresponding functions (slopes of the graphs) indicate the average time (in h) between two cell divisions. ADAR1, adenosine deaminase acting on RNA 1; ADAR1^KO^, fully ADAR1-deficient; DT_50_, division time 50; FITC, fluorescein isothiocyanate; p150^KO^, selectively ADAR1^p150^-deficient; PI, propidium iodide.
(TIF)

S3 Fig Identification and localization of ADAR1-edited sites. (A) Analysis strategy. RNAseq of cellular transcriptome of uninfected, MeV-vac2(GFP)-infected, MeV-C^KO^(GFP)-infected, or IFN A/D–treated HeLa, p150^KO^, and ADAR1^KO^ cells was performed, and variants were detected using GIREMI. ADAR1-specific editing sites were identified through loss of editing in p150^KO^ or ADAR1^KO^ cells. (B) Comparison of total number of variants detected by GIREMI in standard HeLa cells (solid bars) and ADAR1^KO^ cells (hashed bars). Underlying values can be found in S1 Data. (C) Differential variant frequencies in standard HeLa and ADAR1^KO^ cells. Quotient of the absolute number of variants on each chromosome. Higher numbers in HeLa cells than in ADAR1^KO^ cells are indicated by red colors, lower numbers in HeLa cells than in ADAR1^KO^ cells are indicated by blue colors, and no difference is indicated with white. (D) Counts of A>G sites in intergenic regions, UTRs, exons, and introns. Underlying values can be found in S1 Data. (E) Association of A>G sites with retrotransposable elements in HeLa cells. Segments of each circle show the fractions of color-coded types of elements (see legend). Inner circles indicate groups of elements (left column in legend), middle circles indicate subgroups (right column in legend), and outer circles indicate specific elements. Dashed lines are 10% gridlines. Underlying values can be found in S1 Data. ADAR1, adenosine deaminase acting on RNA 1; ADAR1^KO^, fully ADAR1-deficient; GIREMI, Genome-independent Identification of RNA Editing by Mutual Information; IFN A/D, recombinant type-I interferon-alpha; p150^KO^, selectively ADAR1^p150^-deficient; RNAseq, RNA sequencing; UTR, untranslated region.
(TIF)

S4 Fig Distribution of editing sites in ADAR1-edited genes. (A) Distribution of A>G sites in UTRs, exons, and introns of 156 ADAR1-edited transcripts. Underlying values can be found in S1 Data. (B) Distribution of A>G sites in transposable elements (SINE, dark blue; LINE, dark red; no element, gray) within the same transcripts. Underlying values can be found in S1 Data. (C) Comparison of the number of GIREMI-detected A>G sites in 156 ADAR1-edited transcripts expressed in HeLa cells (blue), primary human CSCs (green and orange), and primary human fibroblasts (CTRL, red). The ranks correspond to the gene positions in S1 Table. ADAR1, adenosine deaminase acting on RNA 1; ADAR1^KO^, fully ADAR1-deficient; CSC, cervical stromal cell; GIREMI, Genome-independent Identification of RNA Editing by Mutual Information; LINE, long interspersed nuclear element; SINE, short interspersed nuclear element; UTR, untranslated region.
(TIF)

S5 Fig ADAR1 editing is similar in primary human cells and HeLa cells. (A-E) Editing scores of the ADAR1-edited region in the *VOPP1* 3′ UTR in RNAseq data sets of 5 human donors [38]. (A) healthy donor; (B) AGS1 patient with mutation in *TREX1* gene, (C) AGS2 patient with mutation in *RNASEH2B* gene, (D) AGS4 patient with mutation in *RNASEH2A* gene, (E) AGS5 patient with mutation in *SAMHD1* gene. (F-J) Correlation of editing scores of the *VOPP1* 3′ UTR in primary human samples against HeLa cells. (K) Number of primary human data sets edited by ADAR1 at each nucleotide position. (L) Number of ADAR1-edited sites in HeLa cells found also in the primary data sets. Underlying values can be found in S1 Data. ADAR1, adenosine deaminase acting on RNA 1; AGS1, Aicardi-Goutières Syndrome type 1; AGS2, Aicardi-Goutières Syndrome type 2; AGS4, Aicardi-Goutières Syndrome type 4; AGS5, Aicardi-Goutières Syndrome type 5; RNAseq, RNA sequencing; UTR, untranslated region; *VOPP1*, *vesicular*, *overexpressed in cancer*, *prosurvival protein 1*.
(TIF)

S6 Fig ADAR1-editing in human and macaque *NDUFS1 3′* UTRs. (A) Predicted secondary structure of the human sequence of Fig 2A. (B) Secondary structure of the macaque sequence of Fig 2C. Colored arrows indicate edited Alu repeats shown in Fig 2B. Green numbers and letters refer to approximate positions indicated in Fig 2B. (C) Editing score analysis of macaque *NDUFS1* RNA from heart, kidney, and lung tissue (top to bottom). ADAR1, adenosine deaminase acting on RNA 1; *NDUFS1*, *NADH*:*ubiquinone oxidoreductase core subunit S1*; UTR, untranslated region.
(TIF)

S7 Fig ADAR1-editing of SINE elements is conserved between humans and mice. (A) Coverage plots and transposable elements in the human *BRI3BP* transcript in HeLa and ADAR1^KO^ cells. ADAR1 editing is indicated by green bars. Blue and red boxes below coverage plots indicate location and orientation (blue = positive sense; red = negative sense) of transposable elements. (B) Coverage plots and transposable elements in the *Bri3bp* 3′ UTR of WT and ADAR1-mutant (E861A) C57/BL6 mice [14]. ADAR1 editing is indicated by green bars. Blue and red boxes below coverage plots indicate location and orientation of transposable elements. Colors as in (A). (C and D) Predicted secondary structures of the 3′ UTR of the (C) human *BRI3BP* and (D) murine *Bri3bp* transcripts. ADAR1, adenosine deaminase acting on RNA 1; ADAR1^KO^, fully ADAR1-deficient; SINE, short interspersed nuclear element; UTR, untranslated region; WT, wild-type.
(TIF)

S8 Fig Quantification of ADAR1 editing in the cellular transcriptome. (A) Quantification of top 15,000 most highly expressed transcripts in HeLa, p150^KO^, and ADAR1^KO^ cells UI, virus-infected [MeV-vac2(GFP) or MeV-C^KO^(GFP) at MOI = 3, 24 h post infection], or treated with IFN A/D (1,000 U/ml for 24 h). Heatmap is ordered for highest expression in UI HeLa cells and shows FPKM values from RNAseq analysis. (B) Normalized expression levels of transcripts relative to GAPDH levels. Shown are median levels in the four conditions described in (A). (C) Comparison of ranks of the 16 ADAR1-edited genes identified in this study as well as by Chung and colleagues [34] and Ahmad and colleagues [42]. Underlying values can be found in S1 Data. ADAR1, adenosine deaminase acting on RNA 1; ADAR1^KO^, fully ADAR1-deficient; FPKM, fragments per kilobase of transcript per million mapped reads; GAPDH, glyceraldehyde 3-phosphate dehydrogenase; IFN A/D, recombinant type-I interferon-alpha; MOI, multiplicity of infection; p150^KO^, selectively ADAR1^p150^-deficient; RNAseq, RNA sequencing; UI, uninfected.
(TIF)

S9 Fig Northern blot analysis of viral stocks. RNA extracted from viral stocks (equivalent to 1 × 10^6^ TCID_50_ per lane) was blotted and probed for NT 5–254 of antigenomic [le-N(+), first panel] or genomic [le-N(−), second panel] orientation or NT 15,641–15,890 [L-tr(+), third panel and L-tr(−), fourth panel]. Arrowhead indicates band of full-length genomes/antigenomes (size of 16,728 NT); * indicates band of L mRNA (approximately 6.8 kb); # indicates band of N mRNA (approximately 1.8 kb); & indicates band of N-P bicistronic mRNA (approximately 3.4 kb). Bands in C^KO^(GFP) lanes below 1.8 kb correspond to DI RNA genomes. DI, defective interfering; NT, nucleotide.
(TIF)

S10 Fig RNAseq analysis of ADAR1 editing in MeV genomes. (A) Analysis of viral RNPs purified from infections of HeLa, p150^KO^, and ADAR1^KO^ cells. Left panel: methylene blue staining shows presence of viral genomes and absence of ribosomal RNAs. Center panel: northern blot with single-strand RNA probe recognizing the 5′ end of the MeV (–)-strand genome (L-trailer) confirms presence of full-length genomes. Right panel: northern blot with probe against 18S rRNA shows presence of small amounts of degraded rRNA. (B) RNAseq analysis of RNP preparations. Reads were mapped against MeV-vac2(GFP) (green), human genome 38 (GRCh38p7, blue), and rRNA (red). Bars show percentage of reads mapping to either reference. Underlying values can be found in S1 Data. (C) RNAseq coverage plots of MeV-vac2(GFP) genomes (left panels) or MeV-C^KO^(GFP) genomes (right panels) amplified on standard HeLa (top diagrams), p150^KO^ (middle diagrams), or ADAR1^KO^ cells (bottom diagrams). Total coverage of reads with >95% of nucleotides having a quality score of ≥30 is shown in gray on a logarithmic scale. Coverage plots for reads filtered for >5 (NM > 5, red), >8 (NM > 8, yellow), or >11 mutations (NM > 11, green) are shown on a linear scale. ADAR1, adenosine deaminase acting on RNA 1; ADAR1^KO^, fully ADAR1-deficient; MeV, measles virus; p150^KO^, selectively ADAR1^p150^-deficient; RNAseq, RNA sequencing; RNP, ribonucleocapsid.
(TIF)

S11 Fig In-depth analysis of viral genome editing by ADAR1. (A) Western blot analysis of ADAR1 expression in Vero cells untreated or treated with 1,000 U/ml IFN A/D for 24 h. (B) Proportion of MeV-vac2(GFP) and MeV-C^KO^(GFP) bases with editing scores ≥0.05. Underlying values can be found in S1 Data. (C-D) Neighboring NT frequency analyses of A>G and U>C sites found in (C) MeV-vac2(GFP) genomes and (D) MeV-C^KO^(GFP) genomes amplified in standard HeLa cells. *n* indicates the number of NT positions available for the calculation. Underlying values can be found in S1 Data. ADAR1, adenosine deaminase acting on RNA 1; IFN A/D, recombinant type-I interferon-alpha; NT, nucleotide.
(TIF)

S12 Fig Efficient viral gene expression in Vero cells independently of ADAR1 expression. Infection of Vero and Vero-ADAR1^KO^ cells with MeV-vac2(GFP) or MeV-C^KO^(GFP) at an MOI of 0.1, 32 h post infection. Images show GFP fluorescence (green signal) and phase contrast. Scale bar equals 100 μm. ADAR1, adenosine deaminase acting on RNA 1; ADAR1^KO^, fully ADAR1-deficient; GFP, green fluorescent protein; MOI, multiplicity of infection; UI, uninfected.
(TIF)

S13 Fig Generation and characterization of ADAR1^KO^-MAVS^KO^ and ADAR1^KO^-PKR^KO^ cells. (A) Schematic representation of the human *MAVS* gene, encoding two isoforms, FL-MAVS and ΔMAVS lacking the CARD domain. gRNAs (F and G, black arrowheads) target exon 3, which is only present in the transcript of FL-MAVS. gRNA sequences are underlined in the nucleotide sequence below (PAM in bold letters). Corresponding amino acid sequence of MAVS is indicated on the bottom. Residues comprising the CARD domain are boxed in red. (B) Schematic representation of the human *EIF2AK2* gene encoding PKR. gRNAs (H and I, black arrowheads) target exons 3 and 5, respectively. gRNA sequences are underlined in the nucleotide sequence below (PAM in bold letters). Corresponding amino acid sequence of PKR is shown on the bottom. Residues within RBM I and RBM II are boxed in blue or green, respectively. (C) Western blot analysis of cells transfected with 2.5 μg/ml poly(I:C) for 6 h (+), or untreated cells (−). (D) Quantification of pPKR(T446) signals (top diagram) and pIRF3(S386) signals (bottom diagram) of (C). Values are average ± standard deviation of two independent experiments. Underlying values can be found in S1 Data. ADAR1^KO^, fully ADAR1-deficient; CARD, caspase activation and recruitment domain; FL-MAVS, full-length MAVS; gRNA, guide RNA; MAVS, mitochondrial antiviral signaling protein; PAM, protospacer adjacent motif; pIRF3, phospho-interferon regulatory transcription factor 3; PKR, protein kinase R; pPKR, phospho-PKR; RBM, RNA-binding motif.
(TIF)

S14 Fig Single cycle infection of HeLa clones with recombinant MeV. GFP fluorescence (green) and corresponding phase contrast images of cells infected with MeV-vac2(GFP) (A-D) or MeV-C^KO^(GFP) (E-H) at an MOI of 3. Images were taken 24 h post infection. Scale bar equals 100 μm. (A and E) HeLa cells; (B and F) ADAR1^KO^ cells; (C and G) 5 independent clones of ADAR1^KO^-MAVS^KO^ cells; (D and H) 5 independent clones of ADAR1^KO^-PKR^KO^ cells. ADAR1^KO^, fully ADAR1-deficient; GFP, green fluorescent protein; MAVS, mitochondrial antiviral signaling protein; MeV, measles virus; MOI, multiplicity of infection; PKR, protein kinase R.
(TIF)

S1 Table GIREMI analysis of HeLa cell lines and primary human tissue samples. The table includes the top 156 genes specifically edited by ADAR1 in HeLa cells as identified by the number of A>G editing sites detected by GIREMI. ADAR1, adenosine deaminase acting on RNA 1; GIREMI, Genome-independent Identification of RNA Editing by Mutual Information.
(XLSX)

S1 Data Numerical values of presented diagrams. Each tab contains data values used to generate indicated figure panels.
(XLSX)

## Abbreviations

2′-5′-OAS: 2′-5′-oligoadenylate-synthetase
ADAR1: adenosine deaminase acting on RNA 1
ADAR1^KO^: fully ADAR1-deficient
AGS: Aicardi-Goutières Syndrome
AGS6: AGS type 6
APOBEC: apolipoprotein B mRNA editing enzyme, catalytic polypeptide-like
CARD: caspase activation and recruitment domain
CARDIF: CARD adapter inducing interferon-beta
Cas9: CRISPR-associated 9
Cas9n: Cas9-nickase
CDS: coding sequence
CRISPR: clustered regularly interspaced short palindromic repeat
CSC: cervical stromal cell
DI: defective interfering
DSH1: dyschromatosis symmetrica hereditarian
dsRNA: double-strand RNA
DT_50_: division time 50
FBS: fetal bovine serum
FL-MAVS: full-length MAVS
FPKM: fragments per kilobase of transcript per million mapped reads
GEO: Gene Expression Omnibus
GFP: green fluorescent protein
GIREMI: Genome-independent Identification of RNA Editing by Mutual Information
H.sa.: *Homo sapiens*
IFN: interferon
IFN A/D: recombinant type-I IFN-alpha
IGV: Integrated Genome Viewer
IPS-1: mitochondrial IFN-beta promoter stimulator-1
IRF3: IFN regulatory transcription factor 3
ISG: IFN-stimulated gene
lin. regr.: linear regression
LINE: long interspersed nuclear element
MAVS: mitochondrial antiviral signaling protein
MDA-5: melanoma differentiation–associated gene 5
MeV: measles virus
MeV-C^KO^: MeV unable to express C protein
MFE: minimum free energy
MOI: multiplicity of infection
N: nucleoprotein
N/A: no RNAseq data available because of low or no coverage
NDUFS1: *NADH*:*ubiquinone oxidoreductase core subunit S1*
p150^KO^: selectively ADAR1^p150^-deficient
p150mut_LV_: catalytically inactive ADAR1^p150^
p150wt: catalytically active cytoplasmic isoform
p150wt_LV_: wild-type ADAR1^p150^
Pen/Strep: Penicillin/Streptomycin
PI: propidium iodide
PKR: protein kinase R
poly(I:C): polyinosinic:polycytiylic acid
RBM: RNA-binding motif
RIG-I: retinoic acid–inducible gene I
RLR: RIG-I-like receptor
RNAseq: RNA sequencing
RNP: ribonucleocapsid
SINE: short interspersed nuclear element
SNV: single nucleotide variant
UTR: untranslated region
VISA: virus-induced signaling adapter
VOPP1: *vesicular*, *overexpressed in cancer*, *prosurvival protein 1*
