# Supplementary figures and images for: Extensive editing of cellular and viral double-stranded RNA structures accounts for innate immunity suppression and the proviral activity of ADAR1p150
Source: PLoS Biol. 2018 Nov 29;16(11):e2006577. doi: 10.1371/journal.pbio.2006577 (PMC6264153; doi:10.1371/journal.pbio.2006577)

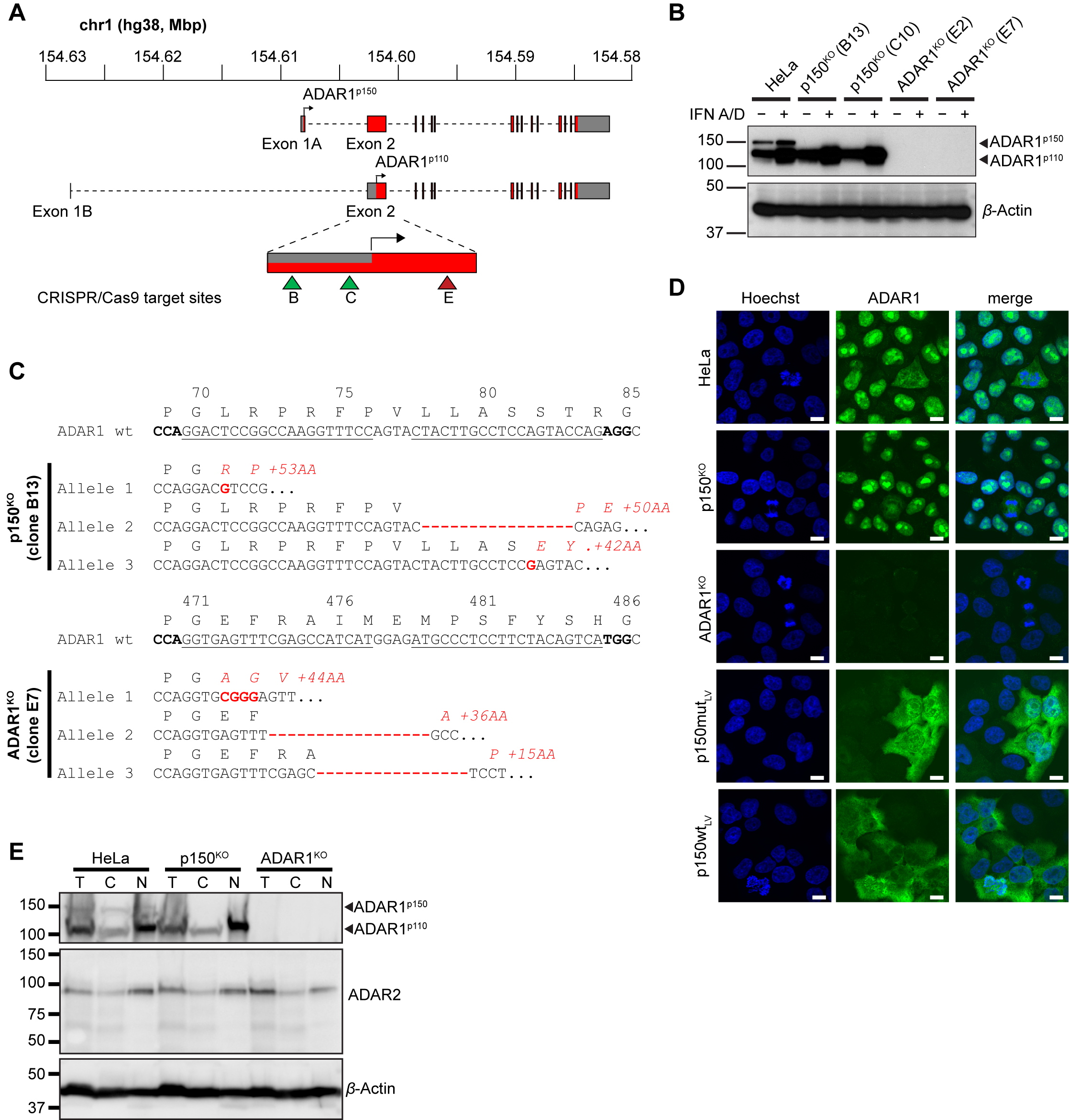

Supplement: S1 Fig — (A) Organization of the ADAR locus on chr1. Transcription from the constitutively active promoter upstream of exon 1B results in translation of the ADAR1p110 isoform from an AUG in exon 2 (M296), indicated by arrow. Transcription from the IFN-inducible promoter upstream of exon 1A results in translation from a start codon within exon 1A (M1), indicated by arrow, giving rise to the ADAR1p150 isoform. Green and red triangles (B, C, E) indicate locations of gRNA binding sites for CRISPR/Cas9 targeting. B and C lead to disruption only of ADAR1p150, whereas E leads to disruption of both isoforms. (B) Genetic characterization of CRISPR/Cas9 disruption of ADAR1p150 in clone B13 and of both isoforms in clone E7. Underlined nucleotides indicate gRNA binding sites; bold nucleotides indicate PAMs. Red highlighted nucleotides indicate insertions or deletions (marked by dashes) causing disruption of ADAR1 open reading frames. Altered amino acids are shown in red above each allele. Three alleles were detected in each clone, indicating that HeLa cells have 3 copies of the ADAR1 locus. (C) Western blot analysis of CRISPR/Cas9-modified HeLa clones deficient for ADAR1p150 (p150KO) or both isoforms (ADAR1KO). Cells were treated with 1,000 U/ml IFN A/D for 24 h or left untreated. Two independent clones for each knock-out are shown. (D) Confocal immunofluorescence staining of HeLa cell clones with altered ADAR1 expression. Nuclear staining (Hoechst) in blue, ADAR1-specific staining in green. Scale bar equals 10 μm. (E) Western blot analysis of total cell extracts (“T”) and cytoplasmic (“C”) and nuclear fractions (“N”) of HeLa, p150KO, and ADAR1KO cells. ADAR1, adenosine deaminase acting on RNA 1; ADAR1KO, fully ADAR1-deficient; Cas9, CRISPR-associated 9; chr1, human chromosome 1; CRISPR, clustered regularly interspaced short palindromic repeat; gRNA, guide RNA; IFN, interferon; IFN A/D, recombinant type-I IFN-alpha; p150KO, selectively ADAR1p150-deficient; PAM, protospacer adjacent moti [file pbio.2006577.s001.tif]

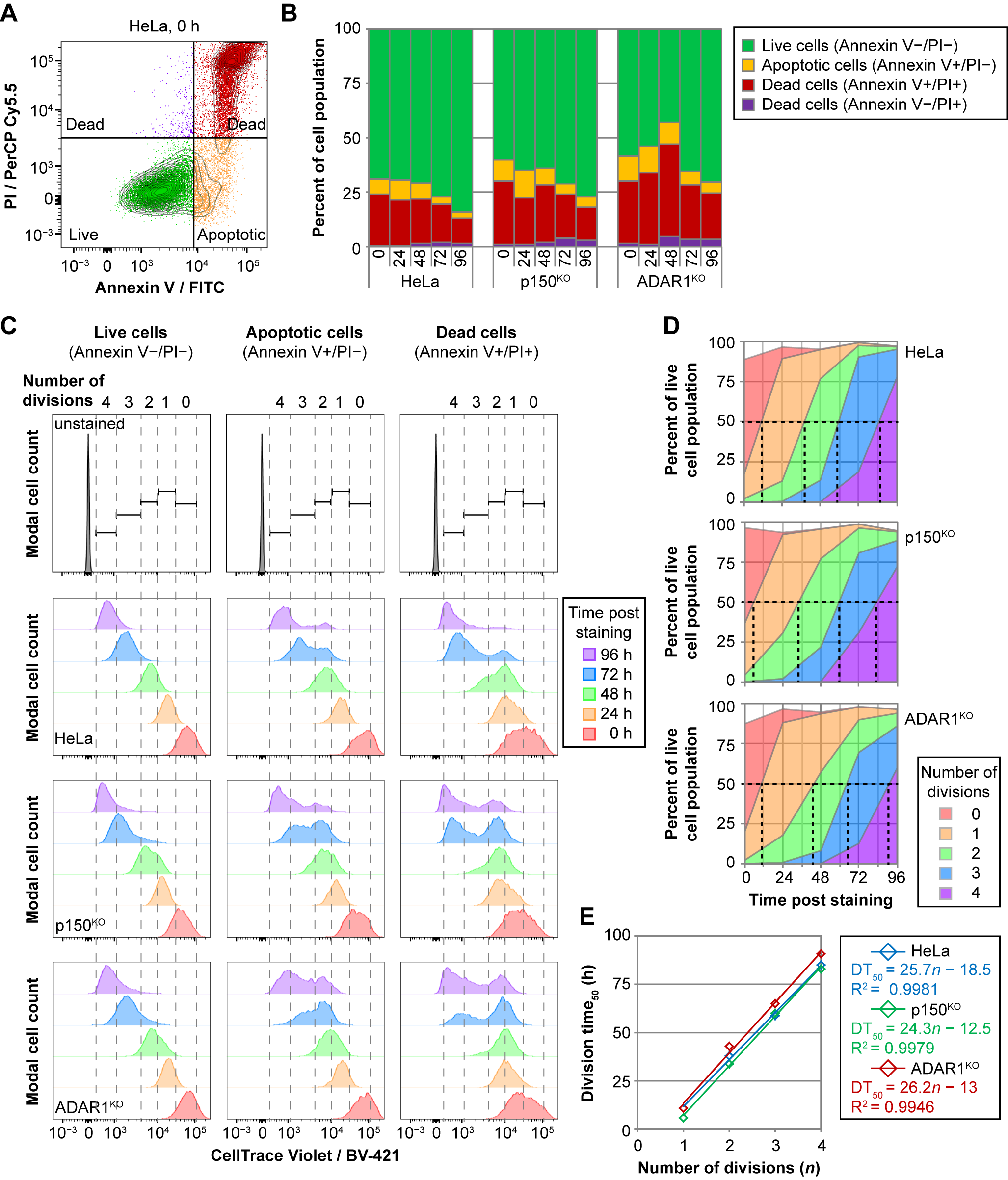

Supplement: S2 Fig — (A) Flow cytometry gating strategy for cell viability. Cells were stained with FITC-conjugated anti-Annexin V for detection of apoptotic cells (x axis) and PI for detection of dead cells (y axis). Single-cell populations were subdivided into live (Annexin V−/PI−), apoptotic (Annexin V+/PI−), and dead cells (Annexin V−/PI+ and Annexin V+/PI+). (B) Quantification of cell viability of HeLa, p150KO, and ADAR1KO cells at various times (in hours) after staining with CellTrace Violet. Underlying values can be found in S1 Data. (C) Analysis of cell division of live (left column), apoptotic (center column), and dead cells (right column) at indicated time points post CellTrace Violet staining. HeLa (second row), p150KO (third row), and ADAR1KO cells (bottom row) were analyzed. Histograms show intensities of CellTrace Violet fluorescence (x axes) and relative cell numbers (modal y axes). Dashed lines indicate gates for 0, 1, 2, 3, and 4 cell divisions based on live HeLa cell signals (second row of panels, left column). (D) Quantification of the percentage of live HeLa (top diagram), p150KO (center diagram), and ADAR1KO cells (bottom diagram) having undergone n divisions at each time point. Black dashed lines indicate time points at which 50% of cells have undergone n divisions (DT50). (E) Extrapolation of DT50 values against number of divisions (n) for HeLa (blue), p150KO (green), and ADAR1KO cells (red). The coefficients of the corresponding functions (slopes of the graphs) indicate the average time (in h) between two cell divisions. ADAR1, adenosine deaminase acting on RNA 1; ADAR1KO, fully ADAR1-deficient; DT50, division time 50; FITC, fluorescein isothiocyanate; p150KO, selectively ADAR1p150-deficient; PI, propidium iodide. (TIF) [file pbio.2006577.s002.tif]

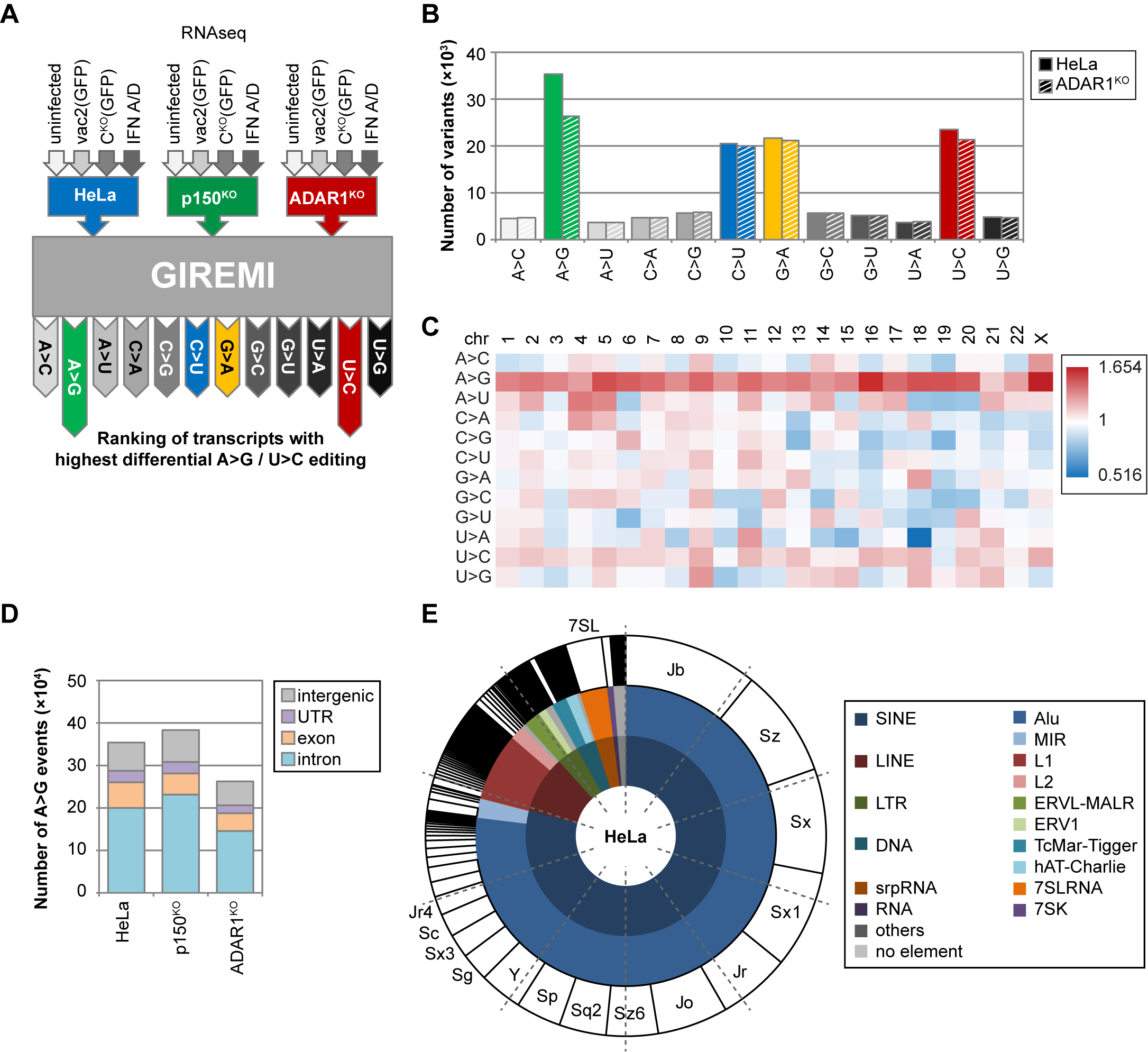

Supplement: S3 Fig — (A) Analysis strategy. RNAseq of cellular transcriptome of uninfected, MeV-vac2(GFP)-infected, MeV-CKO(GFP)-infected, or IFN A/D–treated HeLa, p150KO, and ADAR1KO cells was performed, and variants were detected using GIREMI. ADAR1-specific editing sites were identified through loss of editing in p150KO or ADAR1KO cells. (B) Comparison of total number of variants detected by GIREMI in standard HeLa cells (solid bars) and ADAR1KO cells (hashed bars). Underlying values can be found in S1 Data. (C) Differential variant frequencies in standard HeLa and ADAR1KO cells. Quotient of the absolute number of variants on each chromosome. Higher numbers in HeLa cells than in ADAR1KO cells are indicated by red colors, lower numbers in HeLa cells than in ADAR1KO cells are indicated by blue colors, and no difference is indicated with white. (D) Counts of A>G sites in intergenic regions, UTRs, exons, and introns. Underlying values can be found in S1 Data. (E) Association of A>G sites with retrotransposable elements in HeLa cells. Segments of each circle show the fractions of color-coded types of elements (see legend). Inner circles indicate groups of elements (left column in legend), middle circles indicate subgroups (right column in legend), and outer circles indicate specific elements. Dashed lines are 10% gridlines. Underlying values can be found in S1 Data. ADAR1, adenosine deaminase acting on RNA 1; ADAR1KO, fully ADAR1-deficient; GIREMI, Genome-independent Identification of RNA Editing by Mutual Information; IFN A/D, recombinant type-I interferon-alpha; p150KO, selectively ADAR1p150-deficient; RNAseq, RNA sequencing; UTR, untranslated region. (TIF) [file pbio.2006577.s003.tif]

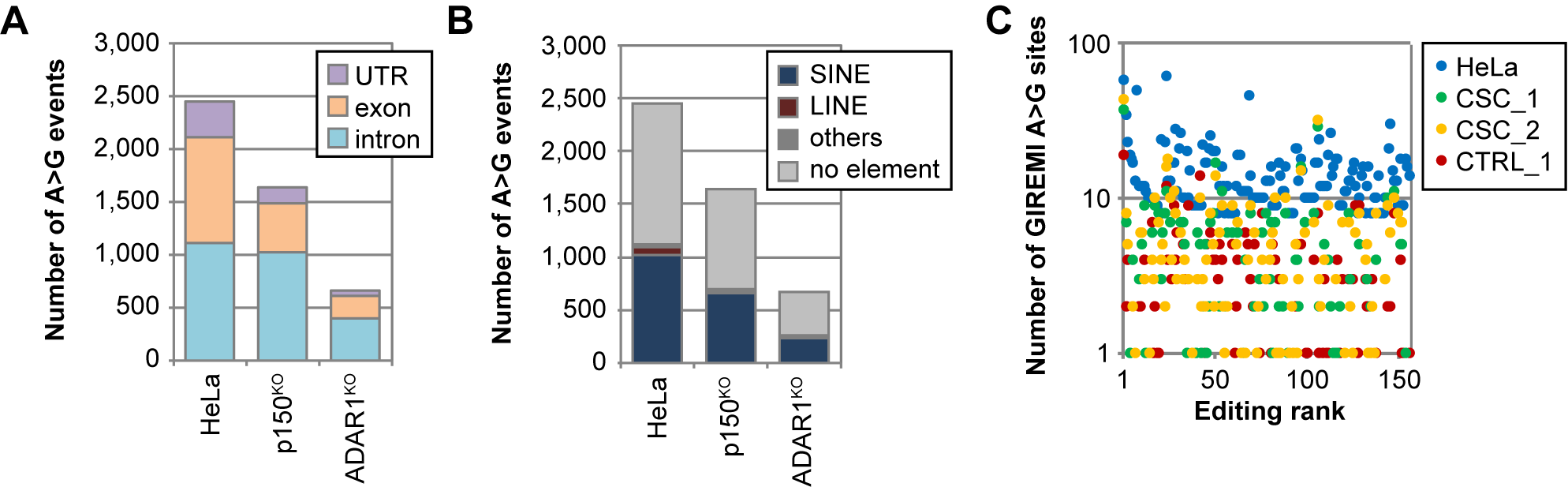

Supplement: S4 Fig — (A) Distribution of A>G sites in UTRs, exons, and introns of 156 ADAR1-edited transcripts. Underlying values can be found in S1 Data. (B) Distribution of A>G sites in transposable elements (SINE, dark blue; LINE, dark red; no element, gray) within the same transcripts. Underlying values can be found in S1 Data. (C) Comparison of the number of GIREMI-detected A>G sites in 156 ADAR1-edited transcripts expressed in HeLa cells (blue), primary human CSCs (green and orange), and primary human fibroblasts (CTRL, red). The ranks correspond to the gene positions in S1 Table. ADAR1, adenosine deaminase acting on RNA 1; ADAR1KO, fully ADAR1-deficient; CSC, cervical stromal cell; GIREMI, Genome-independent Identification of RNA Editing by Mutual Information; LINE, long interspersed nuclear element; SINE, short interspersed nuclear element; UTR, untranslated region. (TIF) [file pbio.2006577.s004.tif]

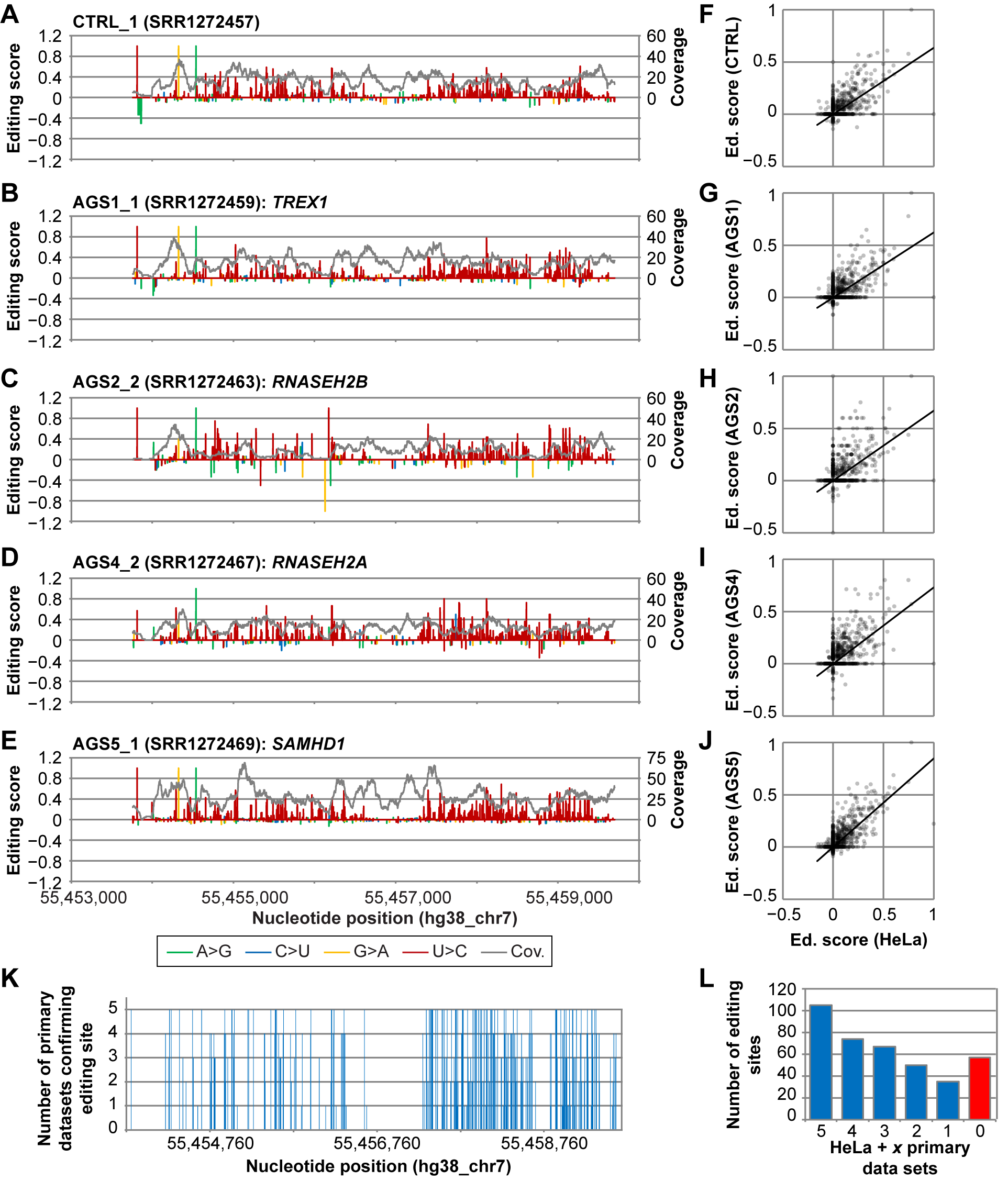

Supplement: S5 Fig — (A-E) Editing scores of the ADAR1-edited region in the VOPP1 3′ UTR in RNAseq data sets of 5 human donors [38]. (A) healthy donor; (B) AGS1 patient with mutation in TREX1 gene, (C) AGS2 patient with mutation in RNASEH2B gene, (D) AGS4 patient with mutation in RNASEH2A gene, (E) AGS5 patient with mutation in SAMHD1 gene. (F-J) Correlation of editing scores of the VOPP1 3′ UTR in primary human samples against HeLa cells. (K) Number of primary human data sets edited by ADAR1 at each nucleotide position. (L) Number of ADAR1-edited sites in HeLa cells found also in the primary data sets. Underlying values can be found in S1 Data. ADAR1, adenosine deaminase acting on RNA 1; AGS1, Aicardi-Goutières Syndrome type 1; AGS2, Aicardi-Goutières Syndrome type 2; AGS4, Aicardi-Goutières Syndrome type 4; AGS5, Aicardi-Goutières Syndrome type 5; RNAseq, RNA sequencing; UTR, untranslated region; VOPP1, vesicular, overexpressed in cancer, prosurvival protein 1. (TIF) [file pbio.2006577.s005.tif]

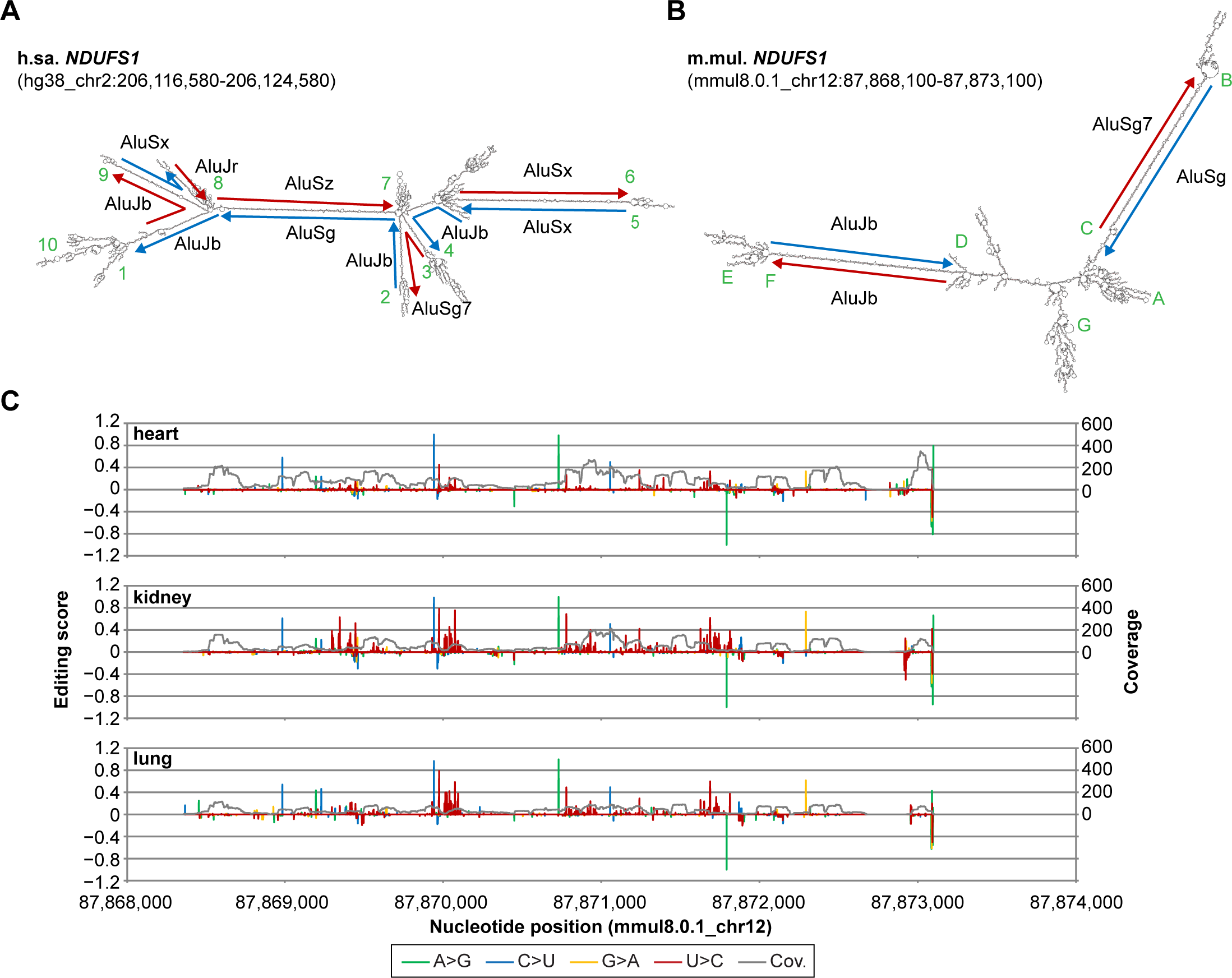

Supplement: S6 Fig — (A) Predicted secondary structure of the human sequence of Fig 2A. (B) Secondary structure of the macaque sequence of Fig 2C. Colored arrows indicate edited Alu repeats shown in Fig 2B. Green numbers and letters refer to approximate positions indicated in Fig 2B. (C) Editing score analysis of macaque NDUFS1 RNA from heart, kidney, and lung tissue (top to bottom). ADAR1, adenosine deaminase acting on RNA 1; NDUFS1, NADH:ubiquinone oxidoreductase core subunit S1; UTR, untranslated region. (TIF) [file pbio.2006577.s006.tif]

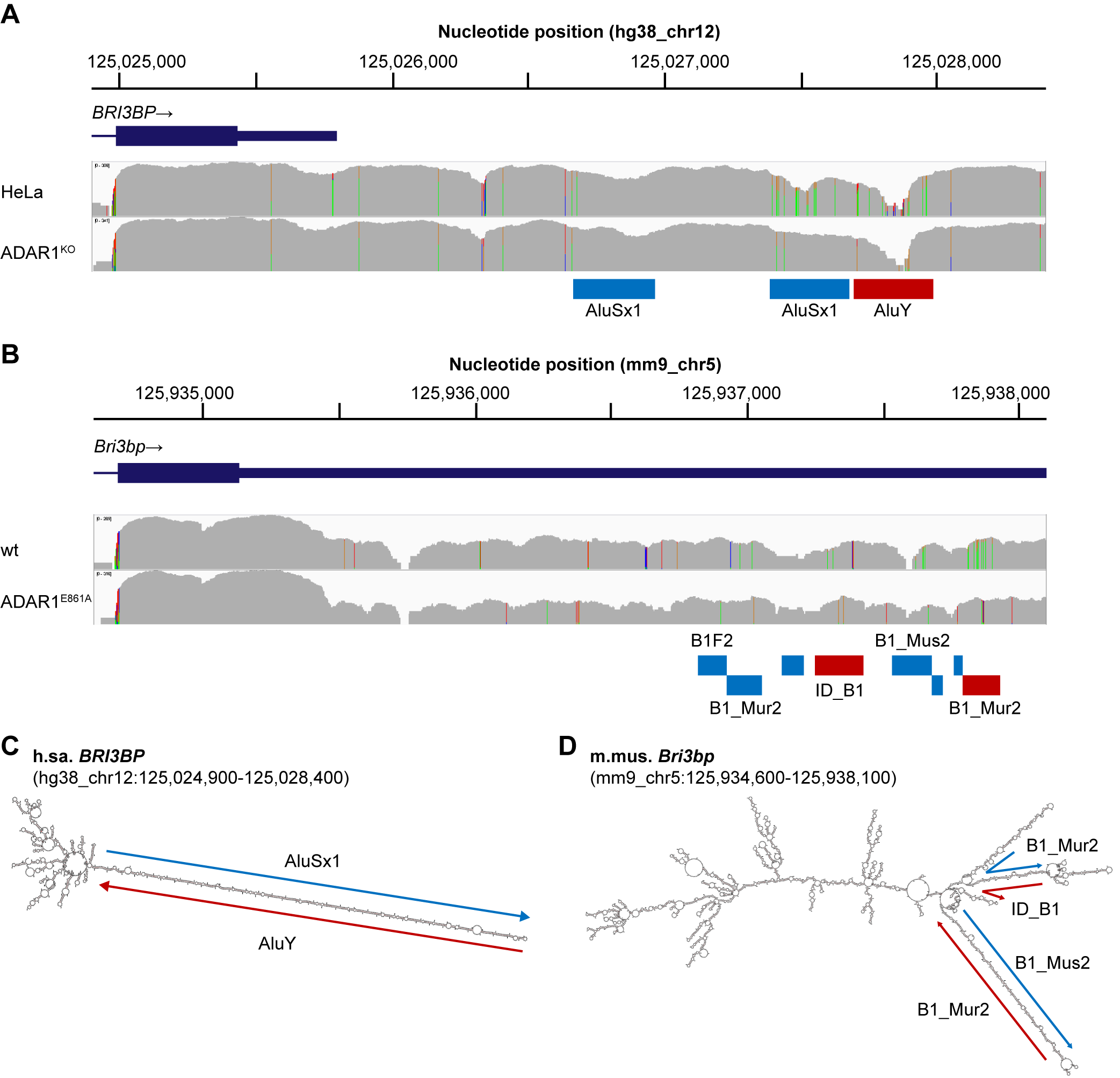

Supplement: S7 Fig — (A) Coverage plots and transposable elements in the human BRI3BP transcript in HeLa and ADAR1KO cells. ADAR1 editing is indicated by green bars. Blue and red boxes below coverage plots indicate location and orientation (blue = positive sense; red = negative sense) of transposable elements. (B) Coverage plots and transposable elements in the Bri3bp 3′ UTR of WT and ADAR1-mutant (E861A) C57/BL6 mice [14]. ADAR1 editing is indicated by green bars. Blue and red boxes below coverage plots indicate location and orientation of transposable elements. Colors as in (A). (C and D) Predicted secondary structures of the 3′ UTR of the (C) human BRI3BP and (D) murine Bri3bp transcripts. ADAR1, adenosine deaminase acting on RNA 1; ADAR1KO, fully ADAR1-deficient; SINE, short interspersed nuclear element; UTR, untranslated region; WT, wild-type. (TIF) [file pbio.2006577.s007.tif]

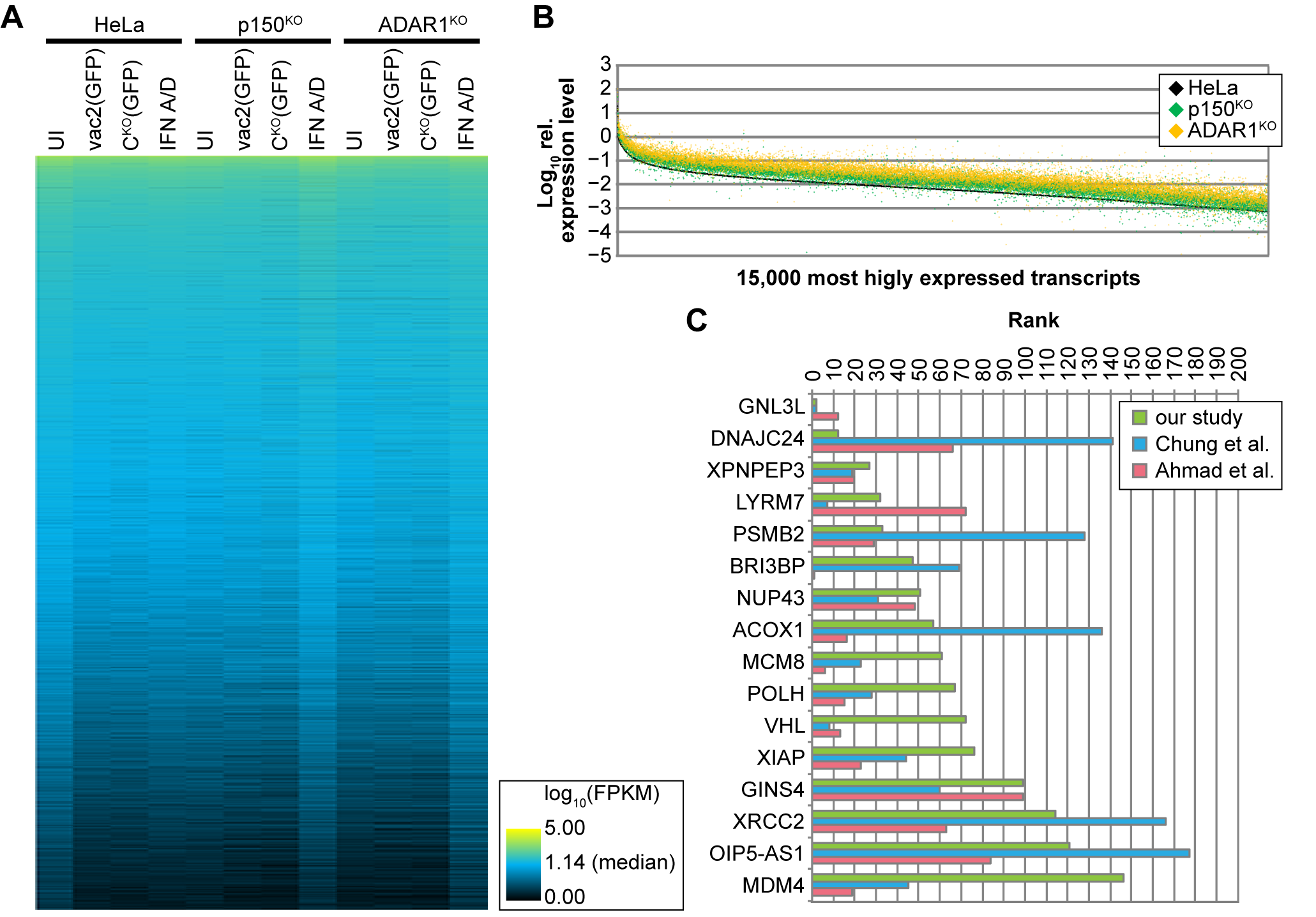

Supplement: S8 Fig — (A) Quantification of top 15,000 most highly expressed transcripts in HeLa, p150KO, and ADAR1KO cells UI, virus-infected [MeV-vac2(GFP) or MeV-CKO(GFP) at MOI = 3, 24 h post infection], or treated with IFN A/D (1,000 U/ml for 24 h). Heatmap is ordered for highest expression in UI HeLa cells and shows FPKM values from RNAseq analysis. (B) Normalized expression levels of transcripts relative to GAPDH levels. Shown are median levels in the four conditions described in (A). (C) Comparison of ranks of the 16 ADAR1-edited genes identified in this study as well as by Chung and colleagues [34] and Ahmad and colleagues [42]. Underlying values can be found in S1 Data. ADAR1, adenosine deaminase acting on RNA 1; ADAR1KO, fully ADAR1-deficient; FPKM, fragments per kilobase of transcript per million mapped reads; GAPDH, glyceraldehyde 3-phosphate dehydrogenase; IFN A/D, recombinant type-I interferon-alpha; MOI, multiplicity of infection; p150KO, selectively ADAR1p150-deficient; RNAseq, RNA sequencing; UI, uninfected. (TIF) [file pbio.2006577.s008.tif]

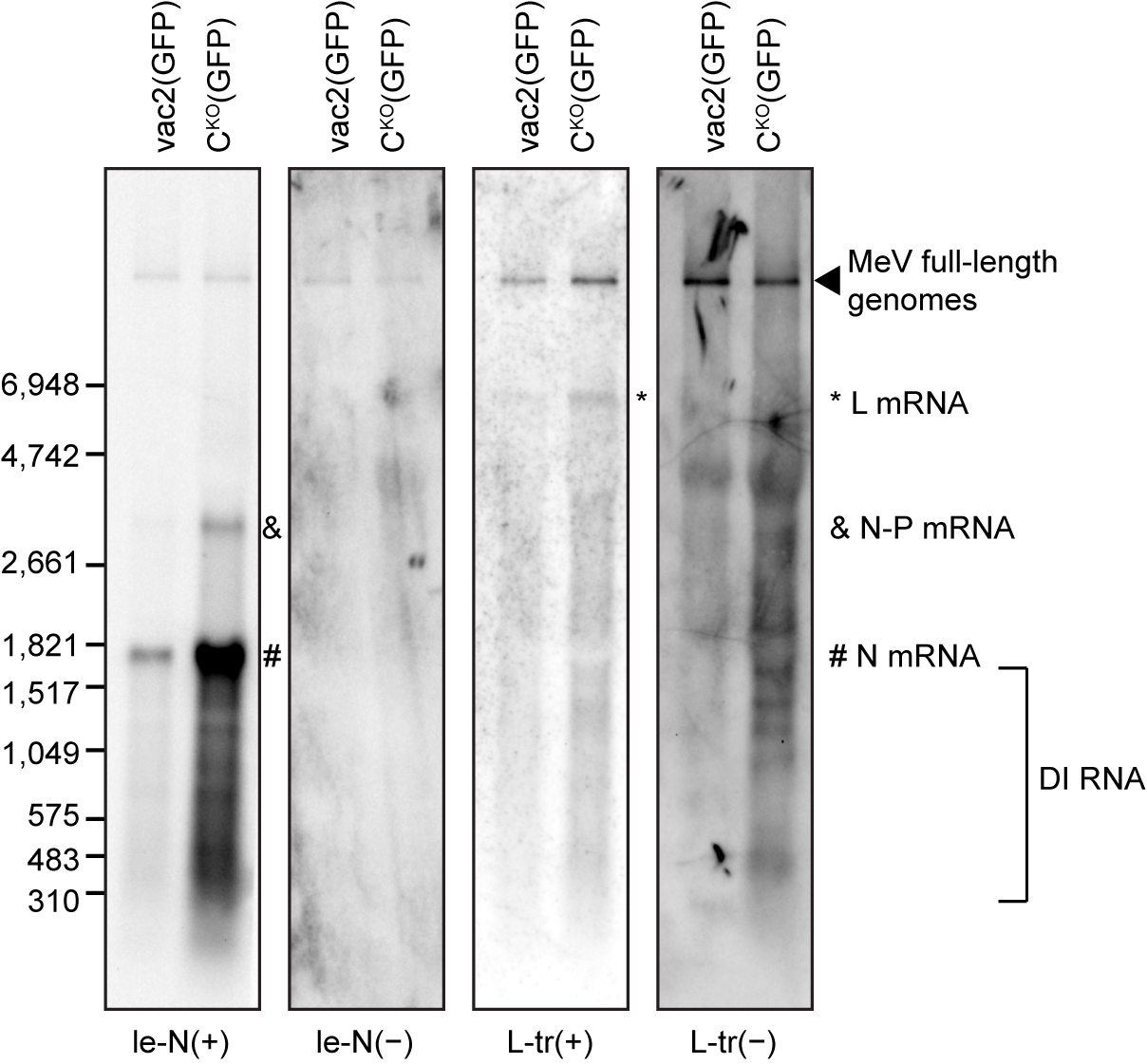

Supplement: S9 Fig — RNA extracted from viral stocks (equivalent to 1 × 10^6^ TCID50 per lane) was blotted and probed for NT 5–254 of antigenomic [le-N(+), first panel] or genomic [le-N(−), second panel] orientation or NT 15,641–15,890 [L-tr(+), third panel and L-tr(−), fourth panel]. Arrowhead indicates band of full-length genomes/antigenomes (size of 16,728 NT); * indicates band of L mRNA (approximately 6.8 kb); # indicates band of N mRNA (approximately 1.8 kb); & indicates band of N-P bicistronic mRNA (approximately 3.4 kb). Bands in CKO(GFP) lanes below 1.8 kb correspond to DI RNA genomes. DI, defective interfering; NT, nucleotide. (TIF) [file pbio.2006577.s009.tif]

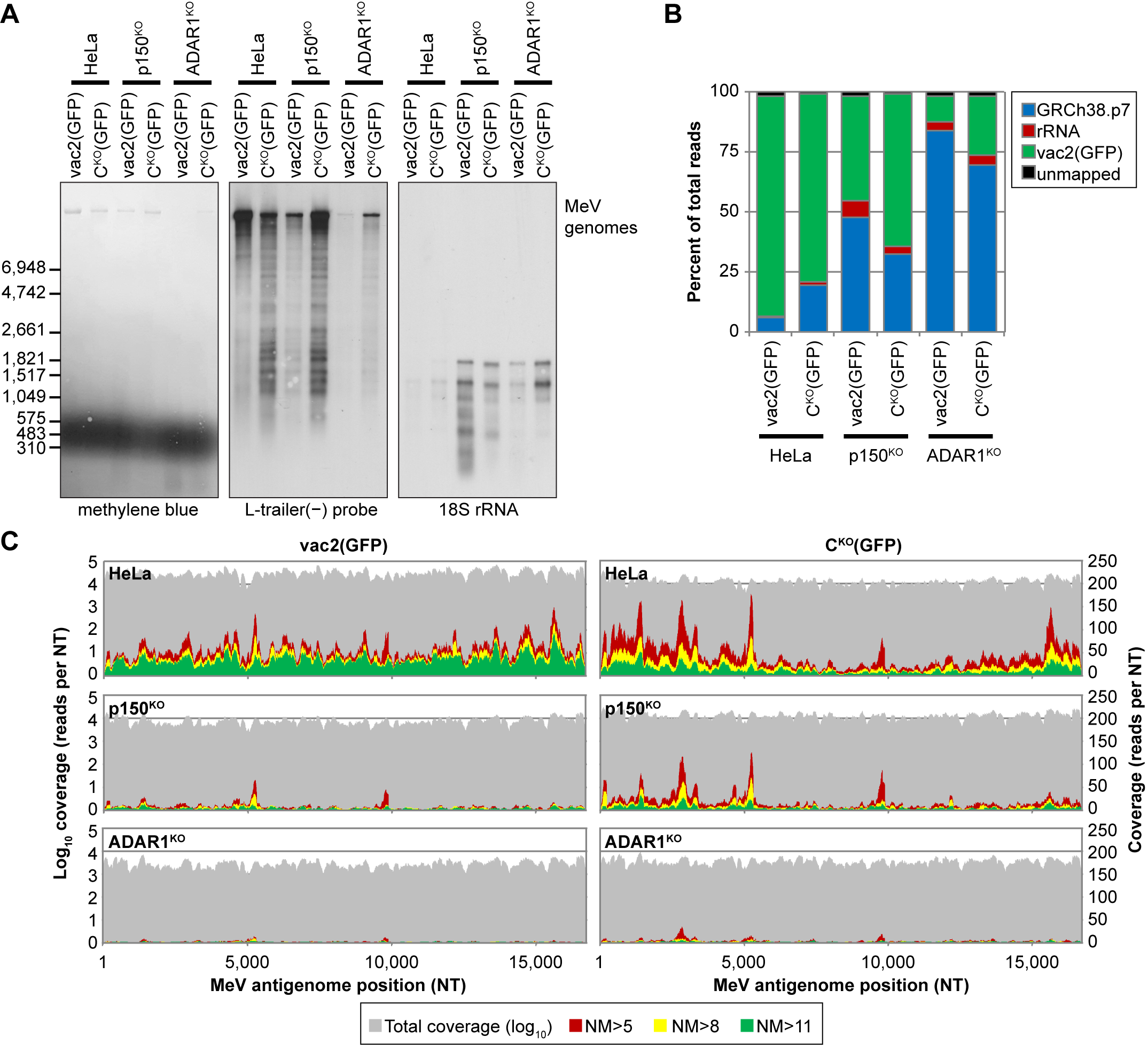

Supplement: S10 Fig — (A) Analysis of viral RNPs purified from infections of HeLa, p150KO, and ADAR1KO cells. Left panel: methylene blue staining shows presence of viral genomes and absence of ribosomal RNAs. Center panel: northern blot with single-strand RNA probe recognizing the 5′ end of the MeV (–)-strand genome (L-trailer) confirms presence of full-length genomes. Right panel: northern blot with probe against 18S rRNA shows presence of small amounts of degraded rRNA. (B) RNAseq analysis of RNP preparations. Reads were mapped against MeV-vac2(GFP) (green), human genome 38 (GRCh38p7, blue), and rRNA (red). Bars show percentage of reads mapping to either reference. Underlying values can be found in S1 Data. (C) RNAseq coverage plots of MeV-vac2(GFP) genomes (left panels) or MeV-CKO(GFP) genomes (right panels) amplified on standard HeLa (top diagrams), p150KO (middle diagrams), or ADAR1KO cells (bottom diagrams). Total coverage of reads with >95% of nucleotides having a quality score of ≥30 is shown in gray on a logarithmic scale. Coverage plots for reads filtered for >5 (NM > 5, red), >8 (NM > 8, yellow), or >11 mutations (NM > 11, green) are shown on a linear scale. ADAR1, adenosine deaminase acting on RNA 1; ADAR1KO, fully ADAR1-deficient; MeV, measles virus; p150KO, selectively ADAR1p150-deficient; RNAseq, RNA sequencing; RNP, ribonucleocapsid. (TIF) [file pbio.2006577.s010.tif]

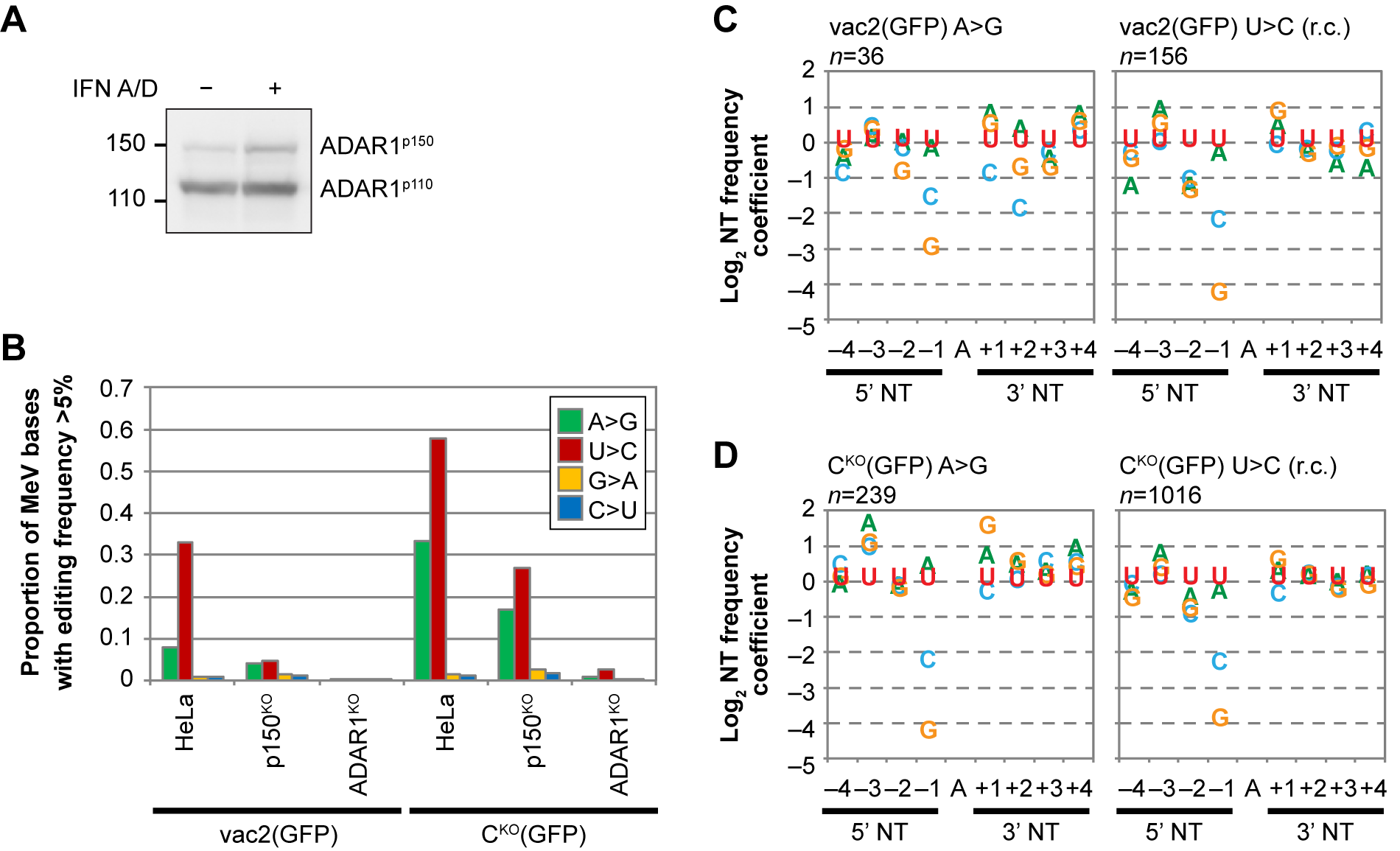

Supplement: S11 Fig — (A) Western blot analysis of ADAR1 expression in Vero cells untreated or treated with 1,000 U/ml IFN A/D for 24 h. (B) Proportion of MeV-vac2(GFP) and MeV-CKO(GFP) bases with editing scores ≥0.05. Underlying values can be found in S1 Data. (C-D) Neighboring NT frequency analyses of A>G and U>C sites found in (C) MeV-vac2(GFP) genomes and (D) MeV-CKO(GFP) genomes amplified in standard HeLa cells. n indicates the number of NT positions available for the calculation. Underlying values can be found in S1 Data. ADAR1, adenosine deaminase acting on RNA 1; IFN A/D, recombinant type-I interferon-alpha; NT, nucleotide. (TIF) [file pbio.2006577.s011.tif]

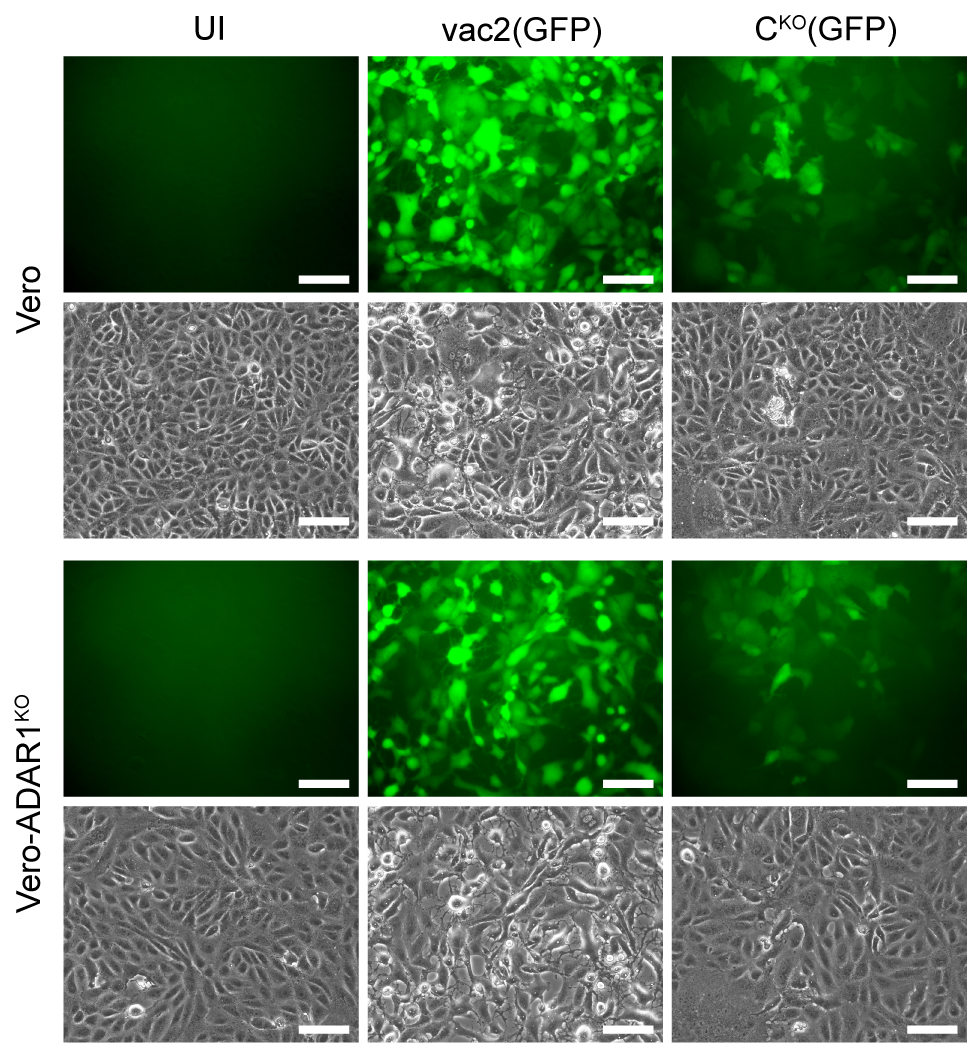

Supplement: S12 Fig — Infection of Vero and Vero-ADAR1KO cells with MeV-vac2(GFP) or MeV-CKO(GFP) at an MOI of 0.1, 32 h post infection. Images show GFP fluorescence (green signal) and phase contrast. Scale bar equals 100 μm. ADAR1, adenosine deaminase acting on RNA 1; ADAR1KO, fully ADAR1-deficient; GFP, green fluorescent protein; MOI, multiplicity of infection; UI, uninfected. (TIF) [file pbio.2006577.s012.tif]

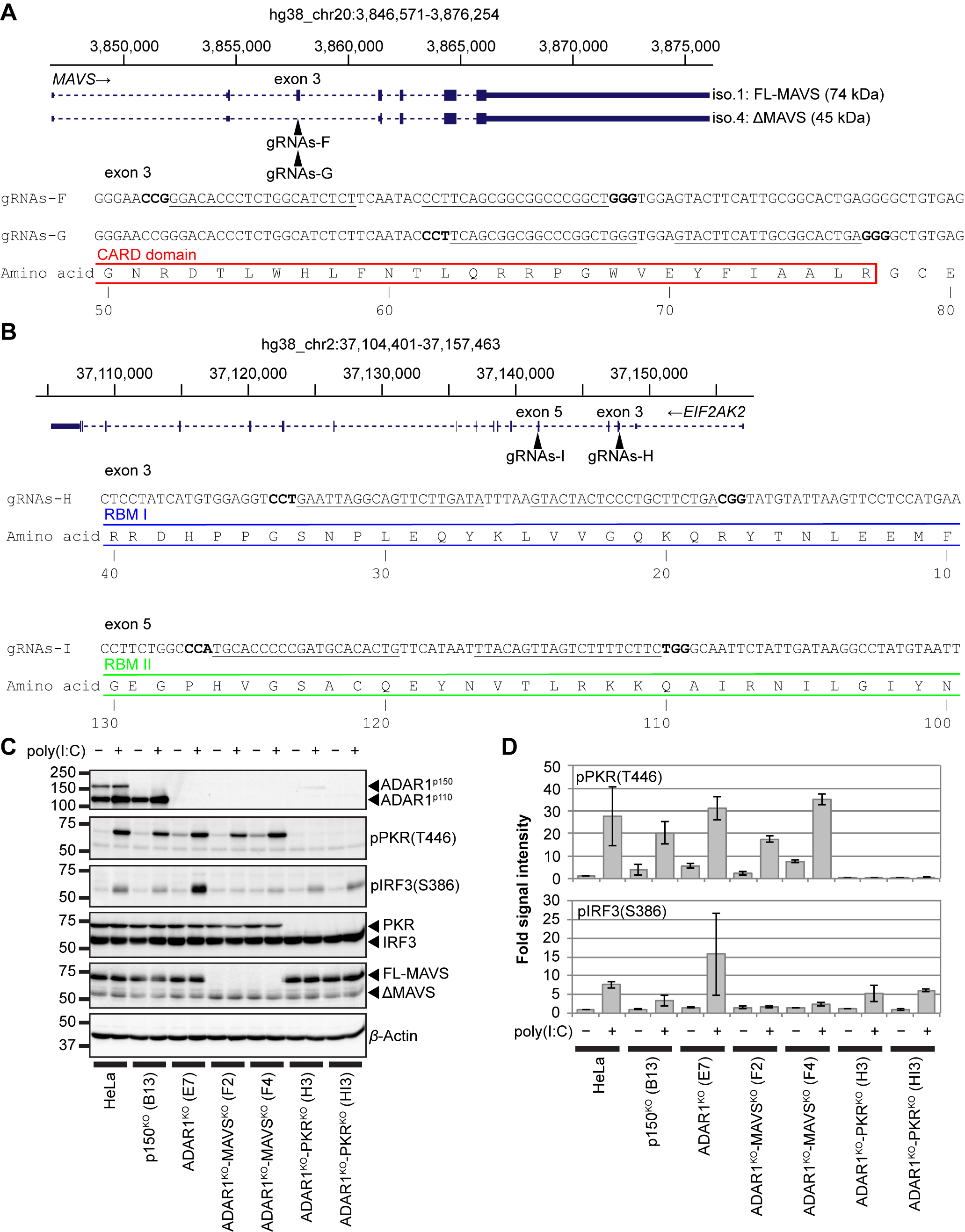

Supplement: S13 Fig — (A) Schematic representation of the human MAVS gene, encoding two isoforms, FL-MAVS and ΔMAVS lacking the CARD domain. gRNAs (F and G, black arrowheads) target exon 3, which is only present in the transcript of FL-MAVS. gRNA sequences are underlined in the nucleotide sequence below (PAM in bold letters). Corresponding amino acid sequence of MAVS is indicated on the bottom. Residues comprising the CARD domain are boxed in red. (B) Schematic representation of the human EIF2AK2 gene encoding PKR. gRNAs (H and I, black arrowheads) target exons 3 and 5, respectively. gRNA sequences are underlined in the nucleotide sequence below (PAM in bold letters). Corresponding amino acid sequence of PKR is shown on the bottom. Residues within RBM I and RBM II are boxed in blue or green, respectively. (C) Western blot analysis of cells transfected with 2.5 μg/ml poly(I:C) for 6 h (+), or untreated cells (−). (D) Quantification of pPKR(T446) signals (top diagram) and pIRF3(S386) signals (bottom diagram) of (C). Values are average ± standard deviation of two independent experiments. Underlying values can be found in S1 Data. ADAR1KO, fully ADAR1-deficient; CARD, caspase activation and recruitment domain; FL-MAVS, full-length MAVS; gRNA, guide RNA; MAVS, mitochondrial antiviral signaling protein; PAM, protospacer adjacent motif; pIRF3, phospho-interferon regulatory transcription factor 3; PKR, protein kinase R; pPKR, phospho-PKR; RBM, RNA-binding motif. (TIF) [file pbio.2006577.s013.tif]

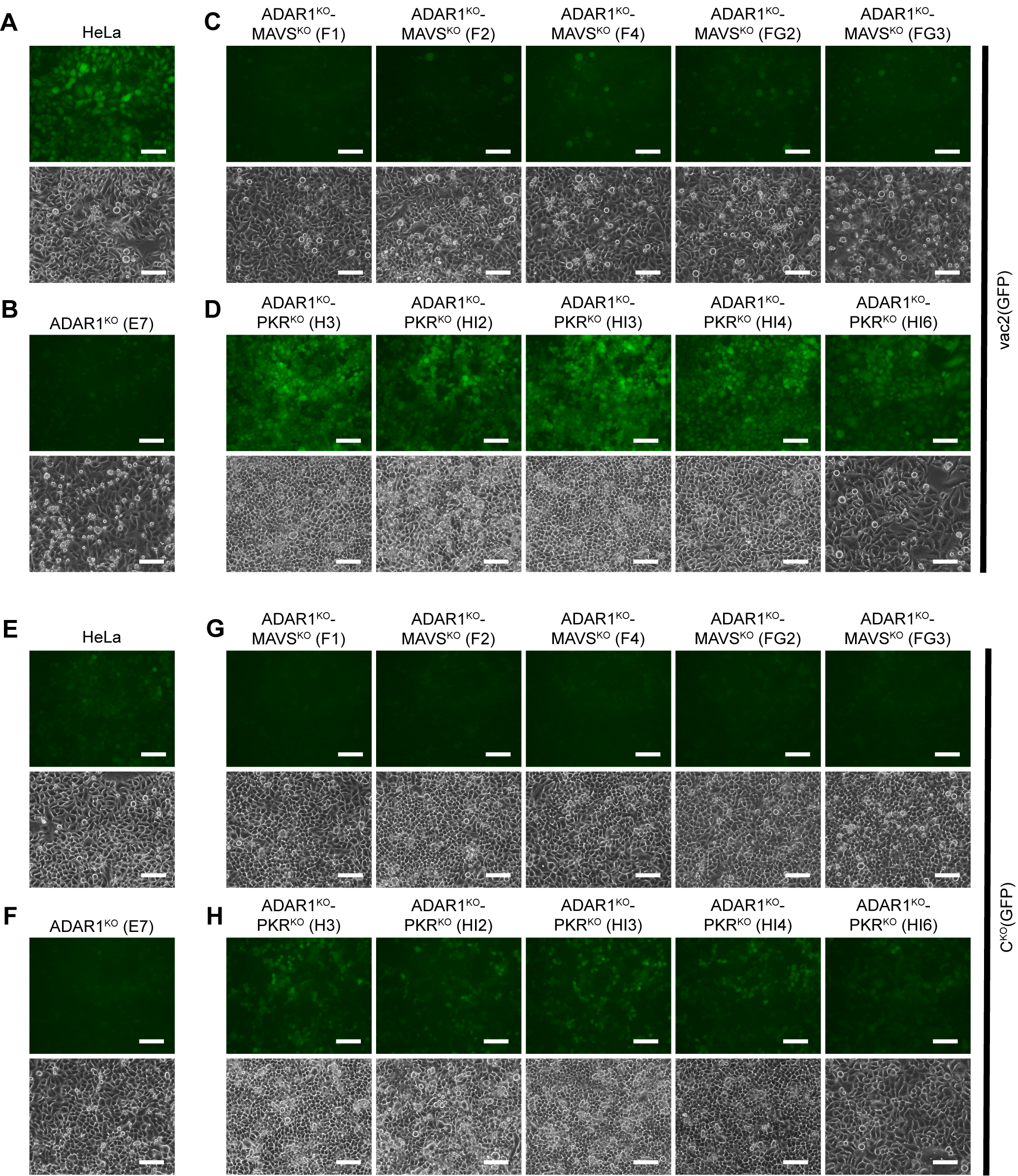

Supplement: S14 Fig — GFP fluorescence (green) and corresponding phase contrast images of cells infected with MeV-vac2(GFP) (A-D) or MeV-CKO(GFP) (E-H) at an MOI of 3. Images were taken 24 h post infection. Scale bar equals 100 μm. (A and E) HeLa cells; (B and F) ADAR1KO cells; (C and G) 5 independent clones of ADAR1KO-MAVSKO cells; (D and H) 5 independent clones of ADAR1KO-PKRKO cells. ADAR1KO, fully ADAR1-deficient; GFP, green fluorescent protein; MAVS, mitochondrial antiviral signaling protein; MeV, measles virus; MOI, multiplicity of infection; PKR, protein kinase R. (TIF) [file pbio.2006577.s014.tif]
